# Trends of overweight, obesity and anthropometric measurements among the adult population in Italy: The CUORE Project health examination surveys 1998, 2008, and 2018

**DOI:** 10.1371/journal.pone.0264778

**Published:** 2022-03-01

**Authors:** Chiara Donfrancesco, Elisabetta Profumo, Cinzia Lo Noce, Daniela Minutoli, Anna Di Lonardo, Brigitta Buttari, Francesca Vespasiano, Serena Vannucchi, Ferruccio Galletti, Graziano Onder, Furio Colivicchi, Daniela Galeone, Paolo Bellisario, Luigi Palmieri

**Affiliations:** 1 Istituto Superiore di Sanità, Rome, Italy; 2 Federico II University of Naples Medical School, Naples, Italy; 3 National Association Hospital Cardiologists-Health Care Foundation, Florence, Italy; 4 U.O.C. Cardiologia Clinica e Riabilitativa, Presidio Ospedaliero San Filippo Neri-ASL Roma 1, Rome, Italy; 5 Italian Ministry of Health, Rome, Italy; Universidad Nacional Autonoma de Mexico, MEXICO

## Abstract

**Background/Objectives:**

Obesity is associated with an increased risk of noncommunicable diseases, such as diabetes, coronary heart disease, stroke, cancers, and conditions, including obstructive sleep apnea and osteoarthritis. Obesity is largely preventable, and halting its rise is one of the World Health Organization Global Action Plan for the Prevention of Noncommunicable Diseases targets. This study aimed to assess trends of anthropometric measurements in Italy using the data collected within the CUORE Project health examination surveys (HESs) 1998, 2008, and 2018.

**Subjects/Methods:**

Within the HESs 1998–2002, 2008–2012, and 2018–2019, anthropometric measurements were collected in random samples of the resident population aged 35–74 years, stratified by age and sex, from 10 Italian Regions in Northern, Central, and Southern Italy (2984 men and 2944 women, 2224 men and 2188 women, 1035 men and 1065 women, respectively). Weight, height, and waist and hip circumferences were measured using standardized methodologies. A standardized questionnaire was used to collect data on education. Indicators were age standardized.

**Results:**

For both men and women, mean body mass index in 2018 was comparable with those in 1998 and 2008 (in 1998, 2008, and 2018—men: 26.7, 27.5, and 27.0 kg/m^2^; women: 26.2, 26.6, and 26.3 kg/m^2^). In 1998, 2008, 2018 prevalence of overweight resulted 49%, 47%, 46% in men and 33%, 32%, 28% in women respectively; prevalence of obesity resulted 17%, 24% 20% in men and 19%, 23%, 23% in women respectively. All indicators of excess weight worsen with increasing age and are more severe in persons with a lower educational level.

**Conclusions:**

Although the overall trend of excess weight over the past two decades appeared to be substantially stable in the Italian adult population, the continuous strengthening of undertaken initiatives should continue since there remains a high proportion of overweight or obesity and a gap between educational levels.

## Background

Most noncommunicable diseases (NCDs) are the result of four common and preventable behaviors, including tobacco use, physical inactivity, unhealthy diet, and harmful use of alcohol, which lead to four key metabolic/physiological changes: raised blood pressure, overweight/obesity, raised blood glucose, and raised cholesterol.

The World Health Organization (WHO) Global NCDs Action Plan 2013–2020 suggested nine global NCD targets in providing a vision for progress by 2025, including halting the rise in obesity [1]. Achieving this target is considered important to reduce NCDs, as obesity is a major risk factor of cardiovascular diseases, diabetes, musculoskeletal disorders, and some cancers and is associated with increased mortality [2–10]. The risk of developing two or more of these diseases, which results in a comorbidity condition, also increases with increasing body weight [11, 12].

Further consistent consequences are related to increased disabilities, reduced productivity, early retirement, reduced length of disability-free healthy living across the life cycle, and increased health care cost [13–15].

Body mass index (BMI) provides the most useful, albeit crude, population-level measure of overweight and obesity, although it does not account for the wide variation in body fat distribution. Other methods, such as waist circumference and waist-to-hip circumferences ratio, besides the measurement of BMI, are considered valuable in identifying the proportion of individuals at increased risk of excess fat-related illnesses [16].

WHO strongly recommends to the Member States the surveillance and monitoring of weight, height, and waist and hip circumference measurements to produce objective indicators that can be compared over time and between geographical areas [1, 17]. The WHO underlines particular attention should be paid to social determinants, in order to monitor health inequality of NCDs and their risk factors—including overweight and obesity—and to create specific health-promoting environments at global, regional and national level [1, 18].

In Italy, the contrast to NCDs is supported by the “Gaining Health: making healthy choices easy” Programme and the National Preventive Plans (NPPs) that were implemented in a context in which NCDs were estimated to account for 91% of all deaths in the period 2000–2016, with proportional mortality from cardiovascular disease of 36% in 2016, and a decreasing trend of premature death since 2000 to 2016 for both men and women aged between 30 and 70 years up to respectively 12% and 7% in 2016 [19].The present study aimed to assess the temporal trends and distribution of anthropometric measurements (height, weight, and waist and hip circumferences) and indicators of excess weight (BMI; waist-to-hip ratio; prevalence of normal weight, overweight, and obesity; high waist-to-hip ratio and abdominal obesity) in the Italian population aged 35–74 years, according to sex, age class, educational level, and region using data measured within the CUORE Project national health examination surveys (HESs) 1998–2002, 2008–2012, and 2018–2019.

## Materials and methods

### Study design

Three HESs were conducted in Italy within the CUORE Project. The first survey was conducted from May 1998 to December 2002 in all Italian regions, enrolling a random sample of 100 men and 100 women aged 35–74 years for every 1.5 million inhabitants (participation rate 50%). The second survey was conducted from March 2008 to July 2012, investigating a sample of 110 men and 110 women aged 35–79 years for every 1.5 million residents in all Italian regions (participation rate 53%). The third survey was conducted from April 2018 to December 2019, in 10 regions (out of 20) chosen in the North, Central, and South Italy, using a sample of 100 men and 100 women aged 35–74 years in each examined region (participation rate 40%).

Within all three HESs, samples were age and sex stratified and randomly selected from the residents’ register with a minimum random samples of 100 men and 100 women enrolled in each involved region, allowing age and sex standardization of results even at regional level.

The three HESs were conducted by the Italian National Institute of Health (Istituto Superiore di Sanità-ISS); the first and second surveys in collaboration with the national scientific association of hospital cardiologists (ANMCO–Associazione Nazionale Medici Cardiologi Ospedalieri) and its foundation [Fondazione per il Tuo Cuore Heart Care Foundation]. Surveys details were published elsewhere [20–22]. The three HESs were approved by the Ethical Committee of the ISS; all participants received an informative note and signed an informed consent. The three HESs are recognized within the Italian National Statistical Program. The second and third surveys were also recognized within the European HES collaboration [23].

### Study procedures and methods

The CUORE Project HESs were implemented to estimate the distribution of NCDs risk factors, conditions at risk and lifestyles. During the HESs, international standardized procedures and methods were used for the data collection and measurements (socio-demographic characteristics, lifestyles, total and HDL cholesterol, triglyceridemia, glycaemia, creatinine, urine albumin, urine creatinine, urine sodium and potassium excretion, respiratory function, bone densitometry, pharmacological treatments, pathological and family history) [20–22].

Participant’s weight and height were measured while they were clothed in their underwear only. A standard electronic scale with a digital display (in the first survey) and a balance beam scale (in the second and third surveys) were used for weight measurements; height was measured using a height ruler. Hip and waist circumferences were measured using a measuring tape while the subject was standing. Waist circumference was measured at the midpoint between the lower edge of the costal arch and the upper edge of the iliac crest while the subjects exhaled. Hip circumference was measured at the largest point of the buttocks. Weight measurements were reported in kilograms and hectograms; height, waist, and hip measurements were reported in centimeters with a decimal (0 or 5). Educational level was investigated through a standardized questionnaire.

### Statistical analysis

The statistical comparison of the 1998–2002, 2008–2012, and 2018–2019 CUORE Project HESs data included 35- to 74-year-old residents in 10 regions, distributed in north, central, and south Italy, involved in all the surveys: Piedmont, Lombardy, Liguria, Emilia Romagna, Tuscany, Lazio, Abruzzo, Basilicata, Calabria, and Sicily.

Weight and height data were used to calculate BMI (weight in kilograms divided by height in square meters). Persons with a BMI < 18.5 kg/m^2^ were considered underweight, 18.5–24.9 kg/m^2^ normal weight, 25.0–29.9 kg/m^2^ overweight, ≥30.0 kg/m^2^ obese, and ≥40.0 kg/m^2^ severely obese [24].

Waist and hip circumferences were used to calculate the prevalence of high waist-to-hip ratio (≥0.90 in men and ≥0.85 in women) and abdominal obesity (waist > 102 cm in men and >88 cm in women) [17].

Educational level was selected as a proxy of socio-economic level; social class was dichotomized as those with primary/middle school (≤8 years, lower education) and high school/university degree (>8 years, higher education).

Mean, standard deviation (SD), and 95% confidence interval of weight, height, BMI, waist and hip circumferences, and prevalence of normal weight, overweight, obesity, high waist-to-hip ratio, and abdominal obesity were assessed by sex, age group (35–44, 45–54, 55–64, and 65–74 years), and periods and, for those with available information, by educational level.

Following the suggestion reported in the WHO Global NCDs Action Plan 2013–2020 [1], indicators, where appropriate, were age standardized using the direct method, referring to the age- and sex-specific distributions of the Italian adult population in 2000, 2010, and 2019 (Italian National Institute of Statistics-ISTAT), for the 1998–2002, 2008–2012, and 2018–2019 HESs respectively [25]. Data were also age standardized using the European Standard Population (EuStPop) 2013 for international comparisons [26]. Because of the small number of underweight and severely obese participants in the sex, age group, and period strata, the corresponding prevalence was not age standardized.

Indicators assessed in the most recent period, 2018–2019, were compared with those of previous periods, 1998–2002 and 2008–2012, through statistical tests and regression models. Associations of the indicators with age and educational level were also determined within periods. Regarding continuous indicators, a t-test was used to assess differences between periods and analysis of variance was used to assess association with age and educational level. Regarding categorical indicators, the chi-square test was used to assess differences between periods and association with age and educational classes. Comparisons between periods were also conducted, adjusting by age and educational level, using linear (continuous indicators) and logistic (categorical indicators) regression models, considering indicators as dependent variables and period (2018–2019/1998–2002 or 2018–2019/2008–2012), age (35–54/55–74 years) and educational level (high/low) as independent variables; the statistical significance of the period was reported. Two-sided p-values <0.05 were considered statistically significant. Statistical analyses were performed using SAS software, release 9.4.

## Results

After the exclusions of persons with missing data for height, weight, and waist or hip circumferences (16 persons in 1998 and nine in 2008); in pregnancy (four in 1998, 19 in 2008, and five in 2018); and with limb in plaster (one in 2018), 2984 men and 2944 women (mean age ± SD: men 55 ± 11, women 54 ± 11), 2224 men and 2188 women (mean age ± SD: men 55 ± 11, women 55 ± 11), and 1035 men and 1065 women (mean age ± SD: men 54 ± 11, women 55 ± 11) were included in the analysis of the 1998–2002, 2008–2012, and 2018–2019 HESs, respectively (S1 Table in S1 File).

### Body mass index

**Fig 1:**
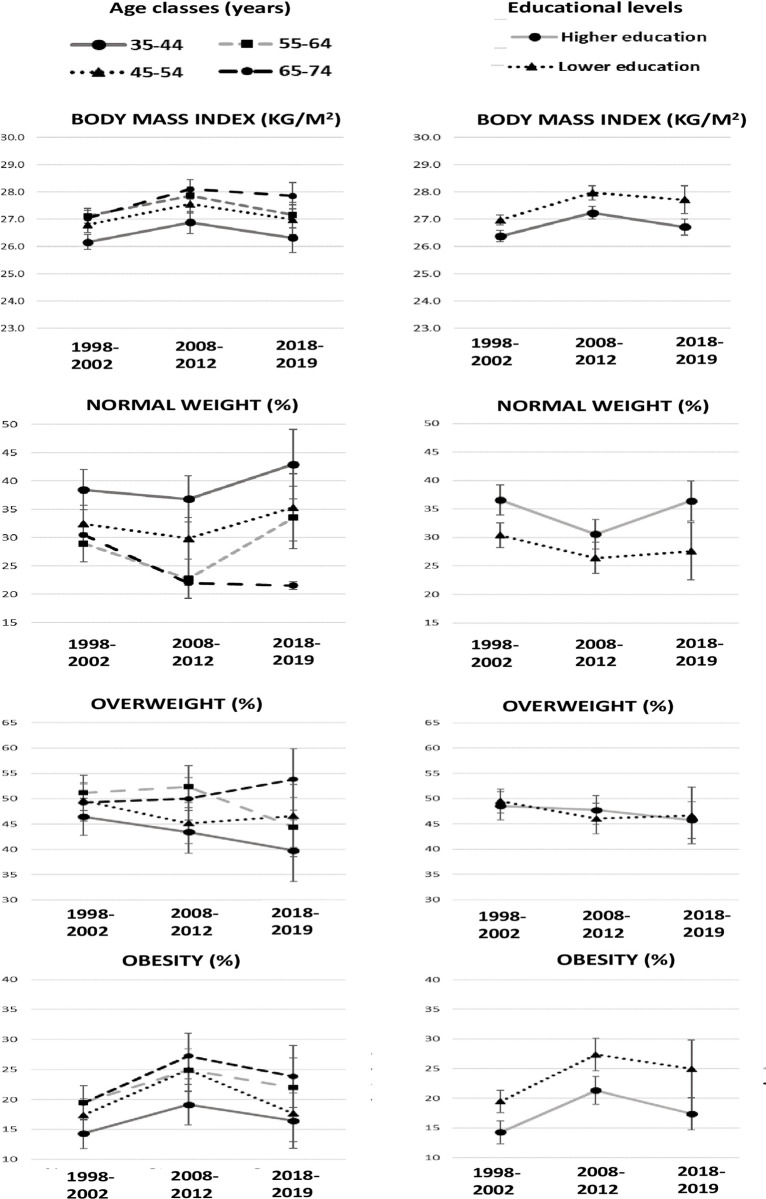
Mean of body mass index and prevalence of normal weight, overweight, and obesity based on measurements, by age class, educational level, and period. Italian resident men aged 35–74 years, the CUORE Project Surveys 1998–2002, 2008–2012, and 2018–2019. Bars refer to 95% confidence intervals. Statistics by educational level were age-standardized using the Italian National Institute of Statistics-ISTAT Italian population 2000, 2010, and 2019, respectively. Higher education—high school or university; lower education—primary or middle school. Underweight: body mass index < 18.5 kg/m^2^. Normal weight: 18.5 ≤ body mass index < 25.0 kg/m^2^. Overweight: 25.0 ≤ body mass index < 30.0 kg/m^2^. Obesity: body mass index ≥ 30.0 kg/m^2^. Pool of the following Italian regions: Piedmont, Lombardy, Liguria, Emilia Romagna, Tuscany, Lazio, Abruzzo, Basilicata, Calabria, and Sicily.

**Fig 2:**
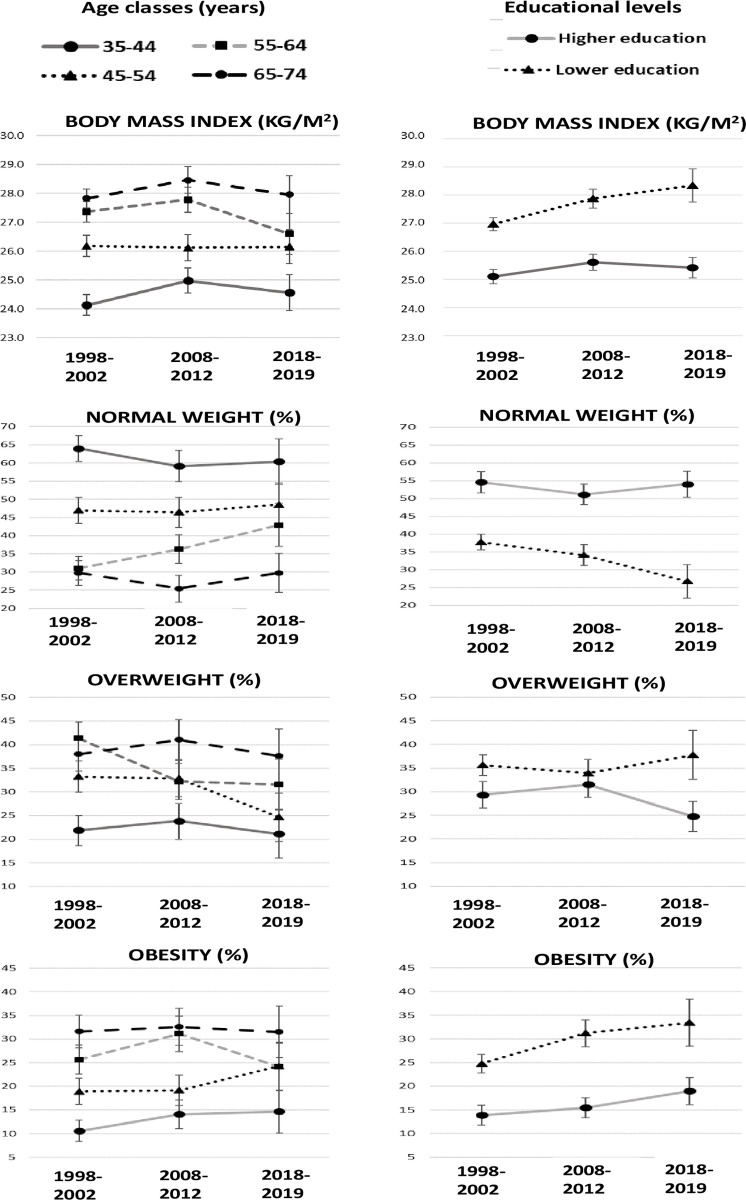
Mean of body mass index and prevalence of normal weight, overweight, and obesity based on measurements, by age class, educational level, and period. Italian resident women aged 35–74 years, the CUORE Project Surveys 1998–2002, 2008–2012, and 2018–2019. Bars refer to 95% confidence intervals. Statistics by educational level were age-standardized using the Italian National Institute of Statistics-ISTAT Italian population 2000, 2010, and 2019, respectively. Higher education—high school or university; lower education—primary or middle school. Underweight: body mass index < 18.5 kg/m^2^. Normal weight: 18.5 ≤ body mass index < 25.0 kg/m^2^. Overweight: 25.0 ≤ body mass index < 30.0 kg/m^2^. Obesity: body mass index ≥ 30.0 kg/m^2^. Pool of the following Italian regions: Piedmont, Lombardy, Liguria, Emilia Romagna, Tuscany, Lazio, Abruzzo, Basilicata, Calabria, and Sicily.

Within the 1998, 2008, and 2018 periods, mean BMI was higher in men than in women and in the overweight class for both sexes as well as in all age groups and educational levels, except for 35- to 44-year-old women, for whom the mean BMI was in the normal weight class (Table 1 and S2–S5 Tables in S1 File and Figs 1 and 2). Within periods, in both men and women, mean BMI progressively increased by age group and was higher in those with a lower level of education, especially for women (S2–S5 Tables in S1 File). The mean BMI difference between educational levels increased progressively in the 1998, 2008, and 2018 periods in both men and women (men: 2%, 3%, and 4%; women: 7%, 8%, and 12%).

**Table 1:** Anthropometric measurements by sex and period (age-adjusted using the Italian population). Italian resident men and women aged 35–74 years, the CUORE Project Surveys 1998–2002, 2008–2012, and 2018–2019.

	**MEN**																	
	**1998–2002**				**2008–2012**				**2018–2019**				**2018–2019 vs 1998–2002**			**2018–2019 vs 2008–2012**		
	***n = 2984***				***n = 2224***				***n = 1035***									
	**mean**	SD	*95% CI*		**mean**	SD	*95% CI*		**mean**	SD	*95% CI*		**Diff**	t-test sign^	Reg sign^^	**Diff**	t-test sign^	Reg sign^^
	** **				** **				** **				** **	** **	** **	** **	** **	** **
**BMI (kg/m** ^**2**^ **)**	**26,7**	3,8	*26*,*6*	*26*,*9*	**27,5**	4,3	*27*,*3*	*27*,*7*	**27,0**	4,2	*26*,*8*	*27*,*3*	**0,3**	*	ns	**-0,5**	**	*
**Height (cm)**	**171,2**	7,1	*170*,*9*	*171*,*4*	**172,0**	6,8	*171*,*7*	*172*,*3*	**173,2**	6,8	*172*,*8*	*173*,*6*	**2,0**	***	***	**1,2**	***	**
**Weight (kg)**	**78,3**	12,3	*77*,*9*	*78*,*8*	**81,4**	13,9	*80*,*9*	*82*,*0*	**81,1**	13,5	*80*,*3*	*81*,*9*	**2,8**	***	***	**-0,3**	ns	ns
**Waist/Hip**	**0,94**	0,06	*0*,*94*	*0*,*94*	**0,96**	0,07	*0*,*96*	*0*,*96*	**0,95**	0,06	*0*,*95*	*0*,*95*	**0,01**	***	**	**-0,01**	**	**
**Waist (cm)**	**94,8**	10,6	*94*,*4*	*95*,*2*	**96,5**	11,7	*96*,*0*	*97*,*0*	**96,0**	11,0	*95*,*3*	*96*,*7*	**1,2**	**	*	**-0,5**	ns	ns
**Hip (cm)**	**101,0**	8,7	*100*,*7*	*101*,*3*	**100,7**	8,8	*100*,*3*	*101*,*0*	**100,9**	7,9	*100*,*4*	*101*,*3*	**-0,1**	ns	ns	**0,2**	ns	ns
	**%**	*95% CI*			**%**	*95% CI*			**%**	*95% CI*			**Diff**	chi-squared sign^	Logistic sign^^	**Diff**	chi-squared sign^	Logistic sign^^
**Normal weight**	**33**	*31*	*35*		**29**	*27*	*31*		**34**	*31*	*37*		**1**	ns	ns	**5**	**	**
**Overweight**	**49**	*47*	*51*		**47**	*45*	*49*		**46**	*43*	*49*		**-3**	ns	*	**-1**	ns	ns
**Obese**	**17**	*16*	*19*		**24**	*22*	*25*		**20**	*17*	*22*		**3**	ns	ns	**-4**	*	ns
**High waist-to-hip ratio**	**80**	*78*	*81*		**82**	*80*	*83*		**78**	*75*	*80*		**-2**	ns	ns	**-4**	**	**
**Abdominal obesity**	**24**	*23*	*26*		**31**	*29*	*33*		**26**	*24*	*29*		**2**	ns	ns	**-5**	**	*
	**WOMEN**																	
	**1998–2002**				**2008–2012**				**2018–2019**				**2018–2019 vs 1998–2002**			**2018–2019 vs 2008–2012**		
	***n = 2944***				***n = 2188***				***n = 1065***									
	**mean**	SD	*95% CI*		**mean**	SD	*95% CI*		**mean**	SD	*95% CI*		**Diff**	t-test sign^	Reg sign^^	**Diff**	t-test sign^	Reg sign^^
	** **		* *		** **		* *		** **		* *							
**BMI (kg/m** ^**2**^ **)**	**26,2**	4,9	*26*,*1*	*26*,*4*	**26,6**	5,3	*26*,*4*	*26*,*9*	**26,3**	5,4	*25*,*9*	*26*,*6*	**0,1**	ns	**	**-0,3**	ns	ns
**Height (cm)**	**158,1**	6,3	*157*,*9*	*158*,*3*	**158,2**	6,3	*157*,*9*	*158*,*4*	**159,0**	6,2	*158*,*7*	*159*,*4*	**0,9**	***	**	**0,8**	**	**
**Weight (kg)**	**65,4**	12,1	*64*,*9*	*65*,*8*	**66,4**	13,1	*65*,*9*	*67*,*0*	**66,3**	13,6	*65*,*5*	*67*,*1*	**0,9**	ns	***	**-0,1**	ns	ns
**Waist/Hip**	**0,84**	0,06	*0*,*83*	*0*,*84*	**0,85**	0,08	*0*,*85*	*0*,*85*	**0,84**	0,07	*0*,*83*	*0*,*84*	**0,00**	ns	ns	**-0,01**	***	***
**Waist (cm)**	**84,2**	11,6	*83*,*8*	*84*,*6*	**86,5**	12,7	*86*,*0*	*87*,*0*	**85,7**	12,9	*84*,*9*	*86*,*5*	**1,5**	**	***	**-0,8**	ns	ns
**Hip (cm)**	**100,5**	10,9	*100*,*1*	*100*,*9*	**101,8**	10,9	*101*,*3*	*102*,*2*	**102,3**	10,8	*101*,*7*	*103*,*0*	**1,8**	***	***	**0,5**	ns	ns
	**%**	*95% CI*			**%**	*95% CI*			**%**	*95% CI*		**Diff**	chi-squared sign^	Logistic sign^^	**Diff**	chi-squared sign^	Logistic sign^^
**Normal weight**	**44**	*43*	*46*		**44**	*42*	*46*		**46**	*43*	*49*		**2**	ns	*	**2**	ns	ns
**Overweight**	**33**	*31*	*35*		**32**	*30*	*34*		**28**	*26*	*31*		**-5**	**	ns	**-4**	*	ns
**Obese**	**21**	*19*	*22*		**23**	*21*	*25*		**23**	*21*	*26*		**2**	ns	***	**0**	ns	ns
**High waist-to-hip ratio**	**44**	*42*	*46*		**49**	*47*	*52*		**41**	*38*	*44*		**-3**	ns	ns	**-8**	***	***
**Abdominal obesity**	**36**	*34*	*38*		**43**	*41*	*45*		**40**	*37*	*43*		**4**	*	***	**-3**	ns	ns

SD: standard deviation; CI: confidence interval; BMI: body mass index; Diff: mean or percentage difference between 2018–2019 and 1998–2002 or 2018–2019 and 2008–2012. Means, standard deviations, and prevalence were age-standardized by Italian National Institute of Statistics-ISTAT Italian population 2000, 2010, and 2019, respectively.

^ t-test to compare variables between periods; chi-square test to compare prevalence between periods.

^^ Linear regression and logistic models were assessed considering indicators as dependent variable and period (2018–2019/1998–2002 or 2018–2019/2008–2012), age (35–54/55–74 years), and educational level (high/low) as independent variables; the statistical significance of the period was reported.

*** p < 0.0001

** p < 0.01

*p < 0.05; ns not significant p-value. Normal weight: 18.5 ≤ body mass index < 25.0 kg/m^2^. Overweight: 25.0 ≤ body mass index < 30.0 kg/m^2^. Obesity: body mass index ≥ 30.0 kg/m^2^. High waist-to-hip ratio: waist-to-hip ratio ≥ 0.90 in men and ≥ 0.85 in women. Abdominal obesity: waist circumference > 102 cm in men and > 88 cm in women. Pool of the following Italian regions: Piedmont, Lombardy, Liguria, Emilia Romagna, Tuscany, Lazio, Abruzzo, Basilicata, Calabria, and Sicily.

The mean BMI in 2018 (1998, 2008, and 2018—men: 26.7, 27.5, 27.0 kg/m^2^; women: 26.2, 26.6, and 26.3 kg/m^2^) was stable compared to that in 2008, with a slight decrease in men; a slight increase occurred in those with lower educational levels when compared to those in 1998 (Table 1 and S2–S5 Tables in S1 File; Figs 1 and 2).

### Height and weight

Expectedly, within the periods, mean height was higher in men than in women by 8% in 1998 and 9% in 2008 and 2018 (Table 1); within the periods, it progressively decreased by age class and was directly associated with educational level (S2–S5 Tables in S1 File).

In men, the 2018 mean height was 1.2 and 2.0 cm higher when compared to those in 2008 and 1998, respectively (by less than 1% in the 35–44 and 65–74 years age classes and by approximately 2% in the 45–64 years age class from 1998 to 2018) (Table 1 and S2 and S4 Tables in S1 File).

In women, the 2018 mean height was 0.9 and 1.0 cm higher when compared to 2008 and 1998, respectively (approximately 1% of increase from 1998 to 2018) (Table 1 and S3 and S5 Tables in S1 File).

Expectedly, within the periods, mean weight was higher in men than in women (Table 1), with a tendency to decrease in men and to increase in women with advancing age and to be higher in those with lower educational level, especially among women (S2–S5 Tables in S1 File).

In men and women, the 2018 mean weight was stable when compared to that in 2008 and slightly increased in men when compared to that in 1998 (Table 1 and S2 and S4 Tables in S1 File).

### Waist and hip circumference and its ratio

Within all three periods and for both men and women, the mean of waist and hip circumferences and their ratio progressively increased by age group (excepted for hip circumference in men in 2018) and was higher in those with a lower level of education (excepted for hip circumference in men) (S2–S5 Tables in S1 File).

The mean of waist and hip circumferences and their ratio in 2018 were stable when compared to those in 2008, with a slight decrease in the waist-to-hip ratio in men and women with higher levels of education, and in 1998, with a slight increase in the waist-to-hip ratio in middle-aged/elderly persons (Table 1 and S2–S5 Tables in S1 File).

### Classes of BMI

Prevalence of underweight was 0.6%, 0.3%, and 0.5% in men and 1.8%, 1.4%, and 2.2% in women in 1998, 2008, and 2018, respectively. Considering the three periods, the prevalence was less than 1% in all age groups in men and from approximately 3% in women aged 35–44 years to 1% in women aged 65–74 years (S6 and S7 Tables in S1 File).

The prevalence of normal weight was higher in women than in men within all the three periods (men: 33%, 29%, and 34%; women 44%, 44%, and 46%), with a relative difference between sexes of 34%, 50%, and 35% in the 1998, 2008, and 2018 surveys, respectively (Table 1).

Within all the three periods, for both men and women, the prevalence of normal weight decreased by age group, and it was directly associated with educational level (S6–S9 Tables in S1 File; Figs 1 and 2).

Prevalence of normal weight in 2018 was higher in men when compared to that in 2008, particularly among those aged 55–64 years and with higher levels of education, and stable in women, except for the less educated with a 7% decrease. Additionally, it was basically stable when compared to that in 1998, with a slight decrease in men aged 65–74 years and in women with higher levels of education (Table 1 and S6–S9 Tables in S1 File; Figs 1 and 2).

**Fig 3:**
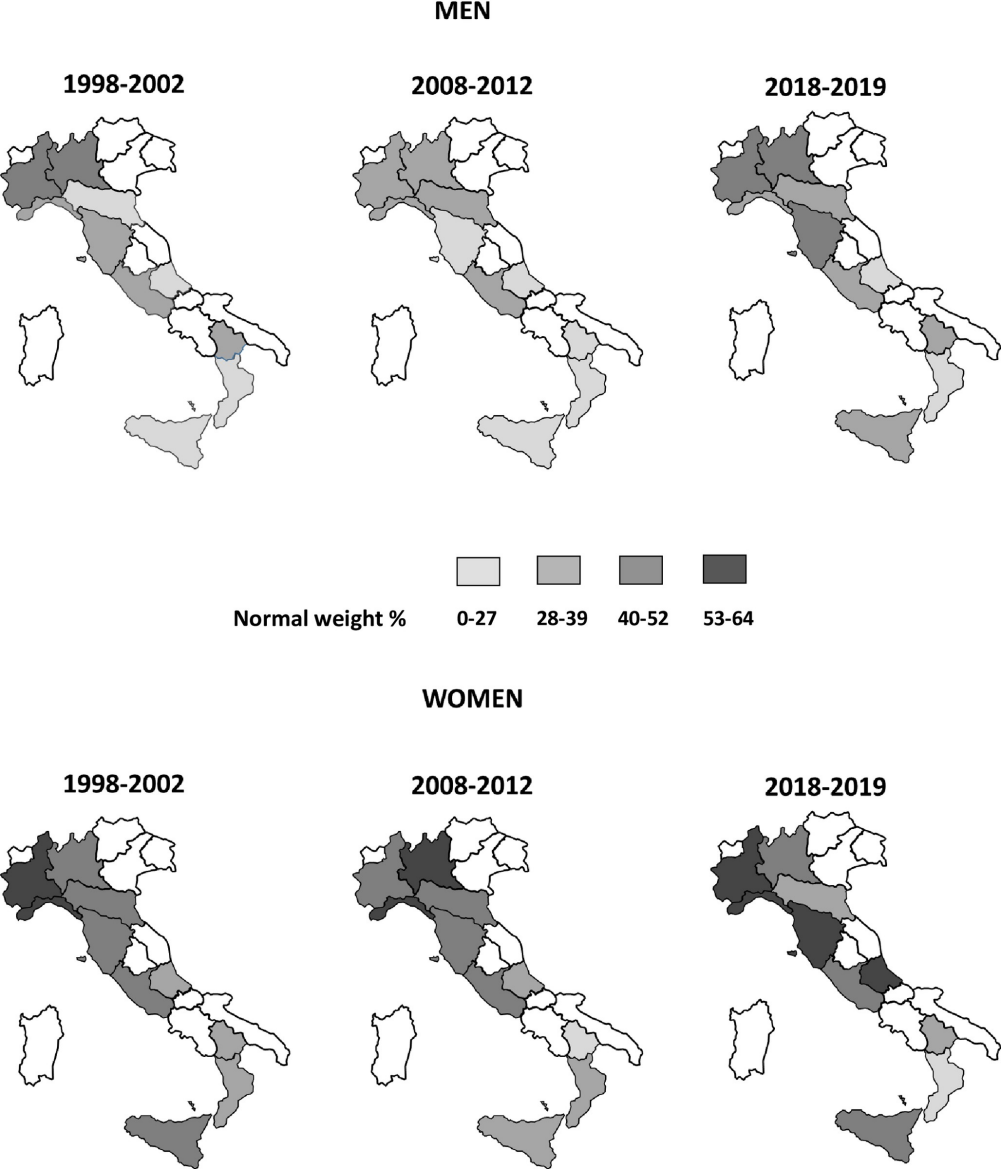
Prevalence of normal weight based on measurements, by sex, regions, and period. Italian resident men and women aged 35–74 years, the CUORE Project Surveys 1998–2002, 2008–2012, and 2018–2019. Prevalence were age-standardized using the National Institute of Statistics-ISTAT Italian population 2000, 2010, and 2019, respectively. Normal weight: 18.5 ≤ body mass index < 25.0 kg/m^2^. Involved Italian regions: Piedmont, Lombardy, Liguria, Emilia Romagna, Tuscany, Lazio, Abruzzo, Basilicata, Calabria, and Sicily.

Normal weight prevalence showed a geographical gradient in favor of the northern regions since 1998; this gradient was confirmed and accentuated in 2018 by the increase of normal weight prevalence in the northern regions; gradient is also present for anthropometric measurements (Fig 3, S10 and S11 Tables in S1 File).

**Fig 4:**
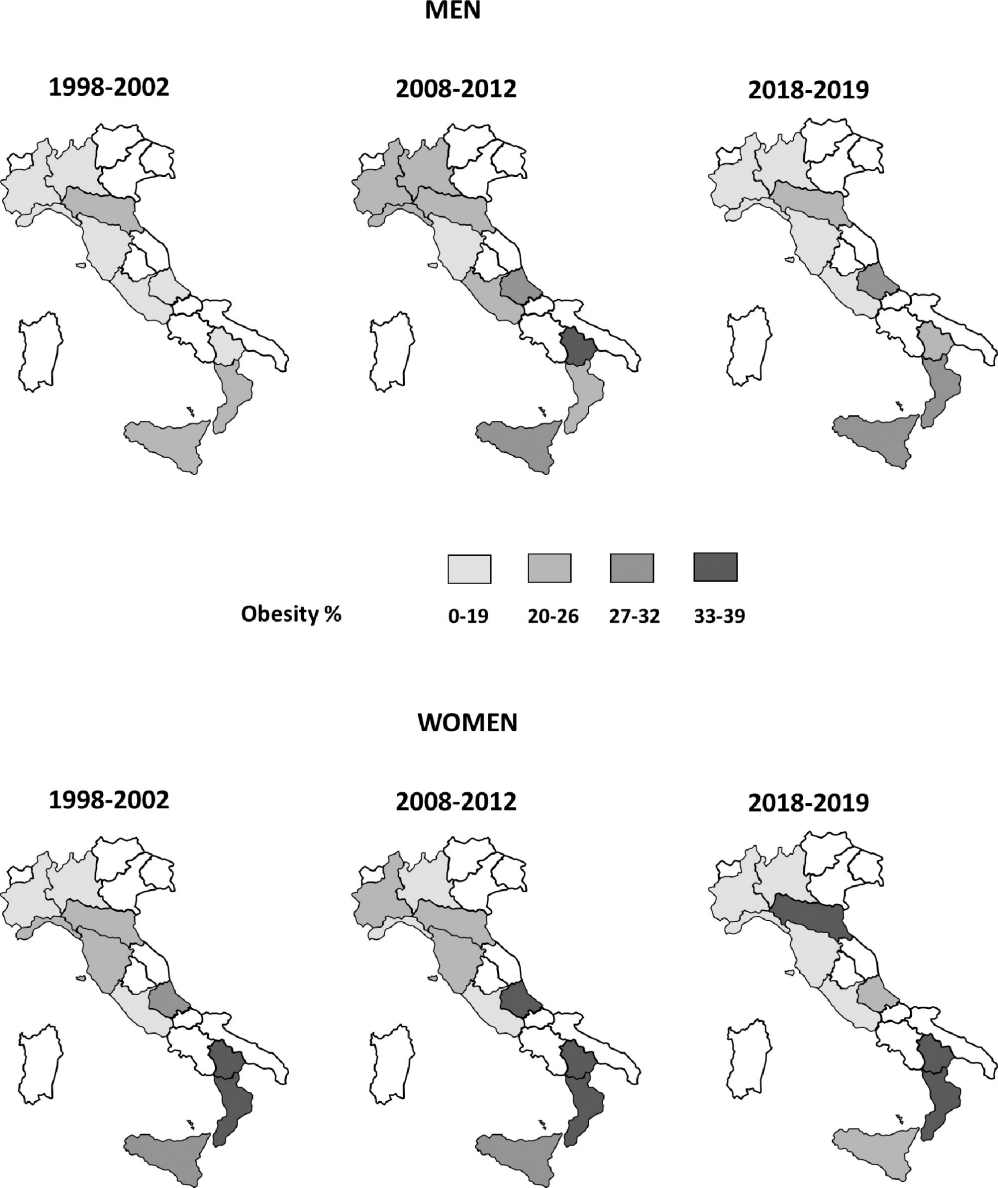
Prevalence of obesity based on measurements, by sex, regions, and period. Italian resident men and women aged 35–74 years, the CUORE Project Surveys 1998–2002, 2008–2012, and 2018–2019. Prevalence were age-standardized using the National Institute of Statistics-ISTAT Italian population 2000, 2010, and 2019, respectively. Obesity: body mass index ≥ 30.0 kg/m^2^. Involved Italian regions: Piedmont, Lombardy, Liguria, Emilia Romagna, Tuscany, Lazio, Abruzzo, Basilicata, Calabria, and Sicily.

Prevalence of overweight was higher in men than in women (1998, 2008, and 2018—men: 49%, 47%, and 46%; women: 33%, 32%, and 28%) with a relative difference between sexes of approximately 50% within all three periods (Table 1). Prevalence of obesity was comparable between men and women (men: 17%, 24%, and 20%; women: 19%, 23%, and 23%) (Table 1). Within all the three periods, the prevalences of both overweight and obesity increased by age group and were directly associated with educational level in women, but only the prevalence of obesity increased in men (S6–S9 Tables in S1 File; Figs 1 and 2). Obesity prevalence appeared to have a geographical gradient with higher values in southern regions since 1998, especially in women (Fig 4, S10 Table in S1 File).

Prevalence of overweight was stable when comparing 2018 to 2008 and 1998, with a decreasing trend over the past 20 years in women with a higher level of education and aged 45–64 years (Table 1 and S8–S11 Tables in S1 File; Figs 1 and 2).

Prevalence of obesity was substantially stable when comparing 2018 to2008, with a slight decrease in men with a higher level of education and women aged 55–64 years; in addition, a substantially stable trend was found when comparing 2018 to 1998, with 5% increase in men with a lower level of education and a slight increase in women when adjusting by educational level (Table 1 and S6–S9 Tables in S1 File; Figs 1 and 2).

Prevalence of severe obesity was 0.4%, 1.3%, and 1.2% in men and 1.9%, 2.2%, and 2.1% in women in 1998, 2008, and 2018, respectively; considering all the three periods and age groups, it varied from 0.0% to 2.4% in men and from 0.0% to 3.9% in women; it was not associated with age groups and tended to increase between 1998 and 2008 in men (S6 and S7 Tables in S1 File).

### Classes of waist and hip circumferences

Prevalence of high waist-to-hip ratio was higher in men than in women (1998, 2008, 2018–men: 80%, 82%, and 78%; women: 44%, 49%, and 41%), with a relative difference between sexes of approximately 50% within all the three periods; however, the prevalence of abdominal obesity was higher in women than in men (1998, 2008, 2018–men: 24%, 31%, 26%; women: 36%, 43%, 40%) (Table 1).

Both in men and women, the prevalence of high waist-to-hip ratio in 2018 was lower when compared to 2008, particularly in those with a higher level of education, middle-aged men, and younger women; it was substantially stable when compared to 1998, with a slight increase in elderly men and a slight decrease in younger men (Table 1 and S6–S9 Tables in S1 File).

The prevalence of abdominal obesity in 2018 was lower in men (particularly in younger class of age and higher education level) and stable in women when compared to 2008, and higher in women and stable in men when compared to 1998 (Table 1 and S6–S9 Tables in S1 File).

Results, age standardized according to the European standard population, are available in S12–S16 Tables in S1 File.

## Discussion

Anthropometric data measured in random samples of the general Italian population aged 35–74 years, recruited in ten Italian regions during three HESs conducted by the ISS in 1998–2002, 2008–2012, and 2018–2019, showed a stable trend over the past one decade for main indicators of excess weight (mean BMI; waist-to-hip ratio and the prevalences of normal weight, overweight, and obesity; high waist-to-hip ratio; and abdominal obesity), with a tendency to increase for normal weight and to decrease for mean BMI and waist-to-hip ratio, and the prevalence of obesity in men with higher educational levels and who are middle-aged, of abdominal obesity in men, and of high waist-to-hip ratio in men and women with higher educational levels.

Stable values were also found in comparison with those of 20 years ago, except for a slight increasing trend for the prevalence of obesity in men with lower educational levels and in women when adjusted for educational level, as well as for abdominal obesity in women, mean BMI in persons with lower education levels, and waist-to-hip ratio mean in middle-aged/elderly persons.

The study evidenced a low proportion of adults in the normal weight class, 34% of men and 46% of women (2018–2019), with approximately one in five adults being obese. All indicators of excess weight worsen with increasing age and are more severe in people with lower educational levels.

To our knowledge, no other national data from 1998 to 2019 were published on burden and temporal trends of antrophometric measurements and several indicators of excess weight based on standardized measurements in random samples of men and women aged 35–74 years considered representative of the general Italian population. Data on antrophometric measurements, such as height, weight, waist and hip circumferences may represent a point of reference at the Italian, European and international level for the study of the evolution of human anthropometry over the generations and its association with biological parameters, such as sex and age, and socio-economic parameters, such as level of education. The reported indicators can contribute to the understanding of the epidemiology of excess weight in Italy and in Europe and in a more general sense in the so-called industrialized countries.

BMI, overweight, and obesity trends are consistent with those of other studies conducted in the Italian adult population. NCD Risk Factor Collaboration, that pooled data of population-based studies with height and weight measurements in adults aged 18 years and older from 200 countries, showed that within the 46 Italian surveys (at community, subnational, and national levels, including the CUORE Project surveys 1998–2002 and 2008–2012), mean BMI and prevalence of overweight did not increase during 1995–2014, and obesity prevalence increased from 1995 to 2016, but not statistically significantly, especially during the period 2005–2016; the latest value of mean BMI and prevalence of overweight and obesity are similar to those of the HES 2018–2019 reported here [27, 28].

Studies conducted in Italy during the period 1983–2004, collecting height and weight by interviewing representative samples of adults, already showed that unfavorable trends in overweight and obesity did not occur in Italian adults [29]. Similar results were found in self-reported data of five Italian surveys conducted annually between 2006 and 2010 in adults [30]. More recently, self-reported data from the Multipurpose Household Survey, an integrated system of cross-sectional social surveys conducted by the ISTAT, collected from 2001 to 2019 in adults, showed that with significant differences among Italian geographical areas, trends of overweight and obesity were increasing moderately up to 2013–2014 and then slightly decreasing without returning to 2001 levels; obesity trend in 20 years under examination was moderate in growth, although not linearly [31, 32]. Another confirmation that the trend during 2008–2019 does not show changes in the percentage of excess weight was provided by the PASSI surveillance of the ISS, which continuously collects self-reported information from the Italian general population (18–69 years) [33, 34]. It is worth pointing out that the excess weight indicators are considerably underestimated in the self-report-based studies [35–38], as a lot of people either overestimate their height or underestimate their weight [15, 39–42].

Additional findings of the present study, including the greater propensity of men to be overweight, the increasing trend of BMI, overweight and obesity with age, and the more severe values in the southern Italy regions and in people with lower educational levels, are consistent with results from other Italian and European Studies [30–34, 43]. The variation of overweight and obesity by age and sex, may be due to the association of age and sex with biological and social causes of overweight and obesity [44]. Socially vulnerable groups widely resulted more affected by excess weight problems probably because they have less access to education and to correct information on lifestyles and health and usually live in areas that do not facilitate an active lifestyle [45]. Very often, moreover, cheaper foods have lower nutritional quality and high energy density, making it difficult to adopt a healthy diet [45, 46].

In the last past two decades, NPPs in Italy were oriented to reduce excess weight problems and related health consequences through intersectional strategies at both population (e.g., facilitating the choice of correct lifestyles—“Gaining Health” Program) and individual levels (e.g., motivational counseling and specific therapeutic groups), promoting, and supporting national surveillance systems and monitoring studies, and through voluntary agreements for salt reduction in food products, school and workplace programs, and public awareness campaigns.

The data here reported may have an important epidemiological impact because they seem to indicate that, although excess weight still affects the majority of the adult population, the right way to stop the epidemic of excess weight in the Italian adult population has been undertaken. Nevertheless, an important impact is also represented by the results showing that in Italy social determinants, such as educational level, still represent a crucial point to be addressed in order to prevent and manage overweight and obesity; similarly to other countries this is more evident in women [45]. Taking into consideration how and when inequities may arise during the life may be important in order to implement a life-course approach (pregnancy, childhood, adulthood, elderly) which includes the prevention of obesity-related health problems [18, 45].

Anyway, although a wide range of policy options has been suggested by the WHO to its members (food and menu labelling, public awareness campaigns, mobile apps, restrictions on food advertising targeting children, school and workplace programmes, and price policies) and a growing number of countries have taken actions to tackle the rise in obesity rate, it has been increasing in almost all European countries since 2000 [15, 46, 47]. Considering self-reported based studies, obesity notably increased from 2000 to 2014 in Finland, France, Ireland, the Netherlands, and Sweden, where obesity rates used to be much lower; considering studies based on measured data, obesity increased from 2006 to 2016 in Finland, Hungary, Luxembourg, and the United Kingdom [41]. Conversely, obesity rates among adults seem to remain relatively stable between 2008 and 2014 in Belgium, Czech Republic, Greece, Latvia, Poland, and Italy and to reach a plateau in 2016 in France and Ireland [41]. Obesity prevalence reported in this study within the 2018–2019 HES was in line with the overall prevalence in other European countries with available measured data in 2018 or nearest years; in such years, Italy seems to be one of the few European countries in which obesity did not substantially increase [15].

Several behavioural and environmental factors have contributed to the long-term rise in obesity rates across European countries, such as the widespread availability of energy-dense foods and an increasingly sedentary lifestyle [18]. These factors have created environments putting people, and especially those in socially disadvantaged groups, more at risk [48]. On the other hand, an increasing number of countries have adopted policies to prevent and reverse obesity. For instance, easy-to-understand interpretative labels were put on the front of prepacked food on a voluntary basis in England, France, Denmark, Norway, Sweden and Lithuania [46]. The taxation of foods high in fat, sugar or salt and/or sugary drinks is also used; at least nine countries in Europe (Belgium, Estonia, Finland, France, Hungary, Ireland, Norway, Portugal and the United Kingdom) have taxes on sugar-sweetened beverages in 2018 [46]. It will be very important to verify the impact and the effectiveness of these measures over time. The burden and temporal trend of overweight and obesity prevalence in each country is the consequence of several factors, including those mentioned above. The 2018 European Commission Reviews of Scientific Evidence and Policies on Nutrition and Physical Activity highlighted the complex and multi-faced nature of excess of weight, and the need to consider a complementary range of actions that are population-wide, integrated, multi-layered, multi-disciplinary and comprehensive, as well as the difficult to identify the most effective combination of interventions due to the quality of available evidence [49]. The need to develop new and innovative evaluation designs and methodologies to support the analysis of the effectiveness and efficiency of a broad range of real-world policies and interventions to promote healthy eating and physical activity and their longer-term impacts and sustainability was also highlighted in other reviews [50, 51].

### Strengths and limitations

Major strengths of this study are the following: the use of standardized procedures and methods to assess anthropometric measurements, allowing objective and reliable estimates of anthropometric indicators at individual and population levels; the good national coverage with the enrolment of the study participants through random age and sex stratification in half of the Italian regions distributed in northern, central, and southern Italy.

Conversely, we acknowledge some study limitations, which should be considered when interpreting results. First, because of the choice of urban districts for the random selection of the study participants within the surveys, the results may not be representative of the habits of the population living in rural areas, even though Italian studies evidenced that, both in men and in women, differences between the urban and rural mean BMI decreased from 1986 up to 2019, reaching zero starting from 2000 [52]. The participation rates in the surveys were lower than desirable, yet consistent, with lower contact rates occurring in more highly urbanized areas and with a decreasing trend of participation observed in HESs in other European countries [53]. The cross-sectional design of the study does not allow to assess causality of the associations between excess weight indicators and educational level. There were differences in the educational level distribution between the three surveys, which is consistent with the increase of secondary and tertiary education assessed in adults from 2008 to 2017 by the Italian National Institute of Statistics [54].

### Conclusions and perspectives

In conclusion, this study demonstrated a substantially stable trend over the past two decades of the main excess weight indicators in three independent samples of the Italian general population aged 35–74 years, conducted in 10 regions approximately 10 years apart from each other (1998, 2008, and 2018). Although this trend in the adult population seems to put Italy on the right path to achieve the WHO Global NCDs Action Plan 2013–2020 target of halting the rise in obesity, it is not possible to lower the attention regarding the problems related to excess weight. In 2018–2019 more than half of men and women were still overweight or obese, with a greater burden in persons with lower educational levels, and with mostly favorable trends found among those with the highest educational level.

Although these results need confirmation through further systematic and periodic monitoring, they have major public health implications and justify the strengthening of initiatives undertaken for the reduction of obesity in Italy since the National Health Plan 1998–2000 and then by the “Gaining Health” Programme and the National Preventive Plans, underlining that attention should be paid to vulnerable groups, such as persons with lower educational levels, thereby reducing disparity, which will be the challenge of the NPP 2020–2025 based on a specific multisector and life-course approach, and of the recent essential levels of assistance, aimed at promoting healthy diet and physical activity.

## Supporting information

S1 File (PDF)
