# Supplementary material for: Trends of overweight, obesity and anthropometric measurements among the adult population in Italy: The CUORE Project health examination surveys 1998, 2008, and 2018
Source: PLoS One. 2022 Mar 1;17(3):e0264778. doi: 10.1371/journal.pone.0264778 (PMC8887738; doi:10.1371/journal.pone.0264778)
Supplement: S1 File — (PDF) [file pone.0264778.s001.pdf]

**Table S1. Demographic characteristics of men and women with available anthropometric measurements within the CUORE Project Surveys 1998–2002, 2008–2012, and 2018–2019.**

| | MEN | | | | | |
|---|---|---|---|---|---|---|
| | 1998-2002 | | 2008-2012 | | 2018-2019 | |
| **Age class (years)** | | | | | | |
| | **n** | **%** | **n** | **%** | **n** | **%** |
| 35-44 | 717 | 24 | 532 | 24 | 249 | 24 |
| 45-54 | 752 | 25 | 589 | 26 | 249 | 24 |
| 55-64 | 784 | 26 | 567 | 25 | 277 | 27 |
| 65-74 | 731 | 24 | 536 | 24 | 260 | 25 |
| **Educational level** | | | | | | |
| | **n** | **%** | **n** | **%** | **n** | **%** |
| Higher education | 1249 | 42 | 1186 | 54 | 728 | 71 |
| Lower education | 1727 | 58 | 1020 | 46 | 304 | 29 |

| | WOMEN | | | | | |
|---|---|---|---|---|---|---|
| | 1998-2002 | | 2008-2012 | | 2018-2019 | |
| **Age class (years)** | | | | | | |
| | **n** | **%** | **n** | **%** | **n** | **%** |
| 35-44 | 711 | 24 | 504 | 23 | 232 | 22 |
| 45-54 | 767 | 26 | 569 | 26 | 272 | 26 |
| 55-64 | 777 | 26 | 584 | 27 | 282 | 26 |
| 65-74 | 689 | 23 | 531 | 24 | 279 | 26 |
| **Educational level** | | | | | | |
| | **n** | **%** | **n** | **%** | **n** | **%** |
| Higher education | 1050 | 36 | 1153 | 53 | 728 | 71 |
| Lower education | 1878 | 64 | 1014 | 47 | 304 | 29 |

Higher education—high school or university; lower education—primary or middle school.
Educational level was reported only for those with available information.
Pool of the following Italian regions: Piedmont, Lombardy, Liguria, Emilia Romagna, Tuscany, Lazio, Abruzzo, Basilicata, Calabria, and Sicily.

**Table S2. Body mass index and measured height, weight, and waist and hip circumference mean levels by age class and period. Italian resident men aged 35–74 years, the CUORE Project Surveys 1998–2002, 2008–2012, and 2018–2019.**

## MEN

### Body mass index (kg/m²)

| Age class (years) | 1998-2002 | | | | ANOVA within period sign | 2008-2012 | | | | ANOVA within period sign | 2018-2019 | | | | ANOVA within period sign | 2018-2019 vs 1998-2002 | | 2018-2019 vs 2008-2012 | |
|---|---|---|---|---|---|---|---|---|---|---|---|---|---|---|---|---|---|---|---|
| | mean | SD | 95% CI | | *** | mean | SD | 95% CI | | *** | mean | SD | 95% CI | | ** | Diff | t-test sign | Diff | t-test sign |
| 35-44 | 26,2 | 3,6 | 25,9 | 26,4 | | 26,9 | 4,7 | 26,5 | 27,3 | | 26,3 | 4,4 | 25,8 | 26,9 | | 0,1 | ns | -0,6 | ns |
| 45-54 | 26,8 | 3,9 | 26,5 | 27,1 | | 27,6 | 4,1 | 27,2 | 27,9 | | 27,0 | 4,4 | 26,4 | 27,5 | | 0,2 | ns | -0,6 | ns |
| 55-64 | 27,1 | 3,8 | 26,9 | 27,4 | | 27,9 | 4,0 | 27,5 | 28,2 | | 27,2 | 4,0 | 26,7 | 27,6 | | 0,1 | ns | -0,7 | * |
| 65-74 | 27,0 | 3,8 | 26,8 | 27,3 | | 28,1 | 4,2 | 27,7 | 28,5 | | 27,9 | 4,0 | 27,4 | 28,3 | | 0,9 | ** | -0,2 | ns |

### Height (cm)

| Age class (years) | 1998-2002 | | | | ANOVA within period sign | 2008-2012 | | | | ANOVA within period sign | 2018-2019 | | | | ANOVA within period sign | 2018-2019 vs 1998-2002 | | 2018-2019 vs 2008-2012 | |
|---|---|---|---|---|---|---|---|---|---|---|---|---|---|---|---|---|---|---|---|
| | mean | SD | 95% CI | | *** | mean | SD | 95% CI | | *** | mean | SD | 95% CI | | *** | Diff | t-test sign | Diff | t-test sign |
| 35-44 | 174,2 | 7,0 | 173,7 | 174,7 | | 174,9 | 6,9 | 174,3 | 175,5 | | 175,1 | 6,3 | 174,3 | 175,9 | | 0,9 | * | 0,2 | ns |
| 45-54 | 171,6 | 7,5 | 171,0 | 172,1 | | 173,1 | 7,0 | 172,5 | 173,6 | | 174,3 | 6,9 | 173,5 | 175,2 | | 2,7 | *** | 1,2 | * |
| 55-64 | 169,2 | 6,9 | 168,8 | 169,7 | | 170,3 | 6,6 | 169,8 | 170,8 | | 173,1 | 7,2 | 172,2 | 174,0 | | 3,9 | *** | 2,8 | *** |
| 65-74 | 167,9 | 7,1 | 167,4 | 168,4 | | 167,5 | 6,8 | 167,0 | 168,1 | | 169,0 | 6,8 | 168,2 | 169,9 | | 1,1 | * | 1,5 | ** |

### Weight (kg)

| Age class (years) | 1998-2002 | | | | ANOVA within period sign | 2008-2012 | | | | ANOVA within period sign | 2018-2019 | | | | ANOVA within period sign | 2018-2019 vs 1998-2002 | | 2018-2019 vs 2008-2012 | |
|---|---|---|---|---|---|---|---|---|---|---|---|---|---|---|---|---|---|---|---|
| | mean | SD | 95% CI | | *** | mean | SD | 95% CI | | *** | mean | SD | 95% CI | | ns | Diff | t-test sign | Diff | t-test sign |
| 35-44 | 79,4 | 12,2 | 78,5 | 80,3 | | 82,3 | 15,5 | 81,0 | 83,6 | | 80,7 | 14,1 | 79,0 | 82,5 | | 1,3 | ns | -1,6 | ns |
| 45-54 | 78,9 | 12,7 | 78,0 | 79,8 | | 82,6 | 13,8 | 81,5 | 83,7 | | 82,1 | 14,5 | 80,3 | 83,9 | | 3,2 | ** | -0,5 | ns |
| 55-64 | 77,8 | 12,4 | 76,9 | 78,6 | | 80,8 | 12,6 | 79,8 | 81,9 | | 81,4 | 12,9 | 79,9 | 82,9 | | 3,6 | *** | 0,6 | ns |
| 65-74 | 76,3 | 11,9 | 75,4 | 77,1 | | 78,9 | 13,1 | 77,8 | 80,1 | | 79,6 | 12,6 | 78,1 | 81,2 | | 3,3 | ** | 0,7 | ns |

### Waist to Hip circumferences ratio

| Age class (years) | 1998-2002 | | | | ANOVA within period sign | 2008-2012 | | | | ANOVA within period sign | 2018-2019 | | | | ANOVA within period sign | 2018-2019 vs 1998-2002 | | 2018-2019 vs 2008-2012 | |
|---|---|---|---|---|---|---|---|---|---|---|---|---|---|---|---|---|---|---|---|
| | mean | SD | 95% CI | | *** | mean | SD | 95% CI | | *** | mean | SD | 95% CI | | *** | Diff | t-test sign | Diff | t-test sign |
| 35-44 | 0,93 | 0,06 | 0,92 | 0,93 | | 0,93 | 0,07 | 0,93 | 0,94 | | 0,92 | 0,06 | 0,92 | 0,93 | | -0,01 | ns | -0,01 | * |
| 45-54 | 0,94 | 0,06 | 0,93 | 0,94 | | 0,96 | 0,08 | 0,95 | 0,96 | | 0,94 | 0,06 | 0,94 | 0,95 | | 0,00 | ns | -0,02 | ** |
| 55-64 | 0,95 | 0,06 | 0,94 | 0,95 | | 0,97 | 0,07 | 0,97 | 0,98 | | 0,96 | 0,06 | 0,95 | 0,97 | | 0,01 | ** | -0,01 | ** |
| 65-74 | 0,95 | 0,06 | 0,94 | 0,95 | | 0,98 | 0,07 | 0,98 | 0,99 | | 0,99 | 0,06 | 0,98 | 1,00 | | 0,04 | *** | 0,01 | ns |

### Waist circumferences (cm)

| Age class (years) | 1998-2002 | | | | ANOVA within period sign | 2008-2012 | | | | ANOVA within period sign | 2018-2019 | | | | ANOVA within period sign | 2018-2019 vs 1998-2002 | | 2018-2019 vs 2008-2012 | |
|---|---|---|---|---|---|---|---|---|---|---|---|---|---|---|---|---|---|---|---|
| | mean | SD | 95% CI | | *** | mean | SD | 95% CI | | *** | mean | SD | 95% CI | | *** | Diff | t-test sign | Diff | t-test sign |
| 35-44 | 92,1 | 10,1 | 91,4 | 92,9 | | 93,3 | 12,4 | 92,3 | 94,4 | | 93,1 | 11,2 | 91,7 | 94,5 | | 1,0 | ns | -0,2 | ns |
| 45-54 | 94,7 | 10,6 | 93,9 | 95,5 | | 96,2 | 11,6 | 95,2 | 97,1 | | 95,2 | 11,5 | 93,8 | 96,6 | | 0,5 | ns | -1,0 | ns |
| 55-64 | 96,3 | 10,7 | 95,5 | 97,0 | | 98,3 | 11,3 | 97,4 | 99,3 | | 96,9 | 10,9 | 95,6 | 98,2 | | 0,6 | ns | -1,4 | ns |
| 65-74 | 97,6 | 11,0 | 96,8 | 98,4 | | 100,2 | 11,4 | 99,3 | 101,2 | | 99,9 | 10,6 | 98,6 | 101,2 | | 2,3 | ** | -0,3 | ns |

### Hip circumferences (cm)

| Age class (years) | 1998-2002 | | | | ANOVA within period sign | 2008-2012 | | | | ANOVA within period sign | 2018-2019 | | | | ANOVA within period sign | 2018-2019 vs 1998-2002 | | 2018-2019 vs 2008-2012 | |
|---|---|---|---|---|---|---|---|---|---|---|---|---|---|---|---|---|---|---|---|
| | mean | SD | 95% CI | | *** | mean | SD | 95% CI | | ** | mean | SD | 95% CI | | ns | Diff | t-test sign | Diff | t-test sign |
| 35-44 | 99,5 | 8,5 | 98,9 | 100,1 | | 99,8 | 9,4 | 99,0 | 100,6 | | 100,7 | 8,4 | 99,7 | 101,8 | | 1,2 | * | 0,9 | ns |
| 45-54 | 100,8 | 8,7 | 100,2 | 101,5 | | 100,5 | 8,5 | 99,8 | 101,2 | | 100,8 | 8,2 | 99,7 | 101,8 | | 0,0 | ns | 0,3 | ns |
| 55-64 | 101,6 | 8,6 | 101,0 | 102,2 | | 101,1 | 8,3 | 100,4 | 101,8 | | 100,9 | 7,3 | 100,0 | 101,7 | | -0,7 | ns | -0,2 | ns |
| 65-74 | 102,9 | 9,0 | 102,3 | 103,6 | | 101,8 | 8,7 | 101,1 | 102,5 | | 101,1 | 7,9 | 100,2 | 102,1 | | -1,8 | ** | -0,7 | ns |

u

SD: standard deviation; CI: confidence interval; BMI: body mass index. ANOVA: Analysis of Variance to compare variables among age classes within periods.t-test was used to compare variables between periods. *** p < 0.0001; ** p < 0.01; *p < 0.05; ns: not significant p-value. 1998–2002 number of men in the 35–44, 45–54, 55–64, and 65–74 age classes (years): 717, 752, 784, and 731. 2008–2012 number of men in the 35–44, 45–54, 55–64, and 65–74 age classes (years): 532, 589, 567, and 536. 2018–2019 number of men in the 35-44, 45-54, 55-64, and 65-74 age classes (years): 249, 249, 277, and 260. Pool of the following Italian regions: Piedmont, Lombardy, Liguria, Emilia Romagna, Tuscany, Lazio, Abruzzo, Basilicata, Calabria, and Sicily.

**Table S3. Body mass index and measured height, weight, and waist and hip circumferences mean levels by age class and period. Italian resident women aged 35–74 years, the CUORE Project Surveys 1998–2002, 2008–2012, and 2018–2019.**

## WOMEN

### Body mass index ($kg/m^2$)

| Age class (years) | 1998-2002 | | | | ANOVA within period *sign* | 2008-2012 | | | | ANOVA within period *sign* | 2018-2019 | | | | ANOVA within period *sign* | 2018-2019 vs 1998-2002 | | 2018-2019 vs 2008-2012 | |
|---|---|---|---|---|---|---|---|---|---|---|---|---|---|---|---|---|---|---|---|
| | mean | SD | *95% CI* | | *** | mean | SD | *95% CI* | | *** | mean | SD | *95% CI* | | *** | Diff | t-test sign | Diff | t-test sign |
| 35-44 | **24,1** | 4,5 | *23,8* | *24,5* | | **25,0** | 5,3 | *24,5* | *25,4* | | **24,6** | 5,1 | *23,9* | *25,2* | | **0,5** | ns | **-0,4** | ns |
| 45-54 | **26,2** | 5,0 | *25,8* | *26,5* | | **26,1** | 5,4 | *25,7* | *26,6* | | **26,2** | 6,0 | *25,4* | *26,9* | | **0,1** | ns | **0,1** | ns |
| 55-64 | **27,4** | 5,2 | *27,0* | *27,7* | | **27,8** | 5,6 | *27,3* | *28,2* | | **26,6** | 5,0 | *26,0* | *27,2* | | **-0,8** | * | **-1,2** | ** |
| 65-74 | **27,8** | 4,8 | *27,5* | *28,2* | | **28,5** | 5,2 | *28,0* | *28,9* | | **28,0** | 5,3 | *27,3* | *28,6* | | **0,2** | ns | **-0,5** | ns |

### Height (cm)

| Age class (years) | 1998-2002 | | | | ANOVA within period *sign* | 2008-2012 | | | | ANOVA within period *sign* | 2018-2019 | | | | ANOVA within period *sign* | 2018-2019 vs 1998-2002 | | 2018-2019 vs 2008-2012 | |
|---|---|---|---|---|---|---|---|---|---|---|---|---|---|---|---|---|---|---|---|
| | mean | SD | *95% CI* | | *** | mean | SD | *95% CI* | | *** | mean | SD | *95% CI* | | *** | Diff | t-test sign | Diff | t-test sign |
| 35-44 | **160,3** | 6,3 | *159,9* | *160,8* | | **160,8** | 6,5 | *160,3* | *161,4* | | **161,4** | 6,2 | *160,6* | *162,2* | | **1,1** | * | **0,6** | ns |
| 45-54 | **158,4** | 6,3 | *158,0* | *158,9* | | **159,2** | 6,1 | *158,7* | *159,7* | | **160,3** | 6,5 | *159,6* | *161,1* | | **1,9** | *** | **1,1** | * |
| 55-64 | **157,0** | 6,1 | *156,6* | *157,5* | | **156,6** | 6,4 | *156,1* | *157,1* | | **158,7** | 5,9 | *158,0* | *159,4* | | **1,7** | *** | **2,1** | *** |
| 65-74 | **155,9** | 6,6 | *155,4* | *156,4* | | **154,7** | 6,2 | *154,1* | *155,2* | | **155,0** | 6,1 | *154,3* | *155,8* | | **-0,9** | ns | **0,4** | ns |

### Weight (kg)

| Age class (years) | 1998-2002 | | | | ANOVA within period *sign* | 2008-2012 | | | | ANOVA within period *sign* | 2018-2019 | | | | ANOVA within period *sign* | 2018-2019 vs 1998-2002 | | 2018-2019 vs 2008-2012 | |
|---|---|---|---|---|---|---|---|---|---|---|---|---|---|---|---|---|---|---|---|
| | mean | SD | *95% CI* | | *** | mean | SD | *95% CI* | | *** | mean | SD | *95% CI* | | * | Diff | t-test sign | Diff | t-test sign |
| 35-44 | **62,0** | 11,9 | *61,1* | *62,9* | | **64,5** | 13,4 | *63,3* | *65,7* | | **64,1** | 14,2 | *62,2* | *65,9* | | **2,1** | * | **-0,4** | ns |
| 45-54 | **65,6** | 12,5 | *64,7* | *66,5* | | **66,1** | 12,9 | *65,0* | *67,1* | | **67,1** | 15,0 | *65,3* | *68,9* | | **1,5** | ns | **1,0** | ns |
| 55-64 | **67,3** | 12,4 | *66,5* | *68,2* | | **68,0** | 13,7 | *66,9* | *69,1* | | **67,0** | 13,0 | *65,5* | *68,5* | | **-0,3** | ns | **-1,0** | ns |
| 65-74 | **67,5** | 11,6 | *66,6* | *68,4* | | **68,0** | 12,2 | *66,9* | *69,0* | | **67,0** | 12,1 | *65,6* | *68,5* | | **-0,5** | ns | **-1,0** | ns |

### Waist to Hip circumferences ratio

| Age class (years) | 1998-2002 | | | | ANOVA within period *sign* | 2008-2012 | | | | ANOVA within period *sign* | 2018-2019 | | | | ANOVA within period *sign* | 2018-2019 vs 1998-2002 | | 2018-2019 vs 2008-2012 | |
|---|---|---|---|---|---|---|---|---|---|---|---|---|---|---|---|---|---|---|---|
| | mean | SD | *95% CI* | | *** | mean | SD | *95% CI* | | *** | mean | SD | *95% CI* | | *** | Diff | t-test sign | Diff | t-test sign |
| 35-44 | **0,82** | 0,06 | *0,81* | *0,82* | | **0,82** | 0,08 | *0,82* | *0,83* | | **0,80** | 0,07 | *0,79* | *0,81* | | **-0,02** | ** | **-0,02** | ** |
| 45-54 | **0,83** | 0,06 | *0,83* | *0,83* | | **0,84** | 0,08 | *0,83* | *0,85* | | **0,82** | 0,06 | *0,82* | *0,83* | | **-0,01** | ns | **-0,02** | ** |
| 55-64 | **0,85** | 0,06 | *0,84* | *0,85* | | **0,86** | 0,08 | *0,85* | *0,87* | | **0,85** | 0,07 | *0,84* | *0,85* | | **0,00** | ns | **-0,01** | ** |
| 65-74 | **0,86** | 0,06 | *0,86* | *0,86* | | **0,88** | 0,08 | *0,88* | *0,89* | | **0,88** | 0,07 | *0,87* | *0,89* | | **0,02** | *** | **0,00** | ns |

### Waist circumferences (cm)

| Age class (years) | 1998-2002 | | | | ANOVA within period *sign* | 2008-2012 | | | | ANOVA within period *sign* | 2018-2019 | | | | ANOVA within period *sign* | 2018-2019 vs 1998-2002 | | 2018-2019 vs 2008-2012 | |
|---|---|---|---|---|---|---|---|---|---|---|---|---|---|---|---|---|---|---|---|
| | mean | SD | *95% CI* | | *** | mean | SD | *95% CI* | | *** | mean | SD | *95% CI* | | *** | Diff | t-test sign | Diff | t-test sign |
| 35-44 | **77,9** | 11,0 | *77,0* | *78,7* | | **81,9** | 12,3 | *80,8* | *83,0* | | **80,3** | 12,8 | *78,6* | *81,9* | | **2,4** | ** | **-1,6** | ns |
| 45-54 | **82,9** | 11,5 | *82,1* | *83,7* | | **85,0** | 12,9 | *84,0* | *86,1* | | **84,4** | 13,7 | *82,8* | *86,0* | | **1,5** | ns | **-0,6** | ns |
| 55-64 | **87,6** | 12,4 | *86,8* | *88,5* | | **89,2** | 13,1 | *88,1* | *90,3* | | **87,5** | 12,4 | *86,0* | *88,9* | | **-0,1** | ns | **-1,7** | ns |
| 65-74 | **90,5** | 11,6 | *89,6* | *91,4* | | **92,0** | 12,6 | *91,0* | *93,1* | | **91,9** | 12,7 | *90,4* | *93,4* | | **1,4** | ns | **-0,1** | ns |

### Hip circumferences (cm)

| Age class (years) | 1998-2002 | | | | ANOVA within period *sign* | 2008-2012 | | | | ANOVA within period *sign* | 2018-2019 | | | | ANOVA within period *sign* | 2018-2019 vs 1998-2002 | | 2018-2019 vs 2008-2012 | |
|---|---|---|---|---|---|---|---|---|---|---|---|---|---|---|---|---|---|---|---|
| | mean | SD | *95% CI* | | *** | mean | SD | *95% CI* | | *** | mean | SD | *95% CI* | | *** | Diff | t-test sign | Diff | t-test sign |
| 35-44 | **95,3** | 10,7 | *94,6* | *96,1* | | **99,4** | 10,9 | *98,4* | *100,3* | | **99,8** | 10,6 | *98,5* | *101,2* | | **4,5** | *** | **0,4** | ns |
| 45-54 | **99,7** | 11,1 | *99,0* | *100,5* | | **100,9** | 10,6 | *100,0* | *101,8* | | **102,2** | 11,8 | *100,8* | *103,6* | | **2,3** | ** | **1,3** | ns |
| 55-64 | **103,3** | 11,2 | *102,5* | *104,0* | | **103,6** | 11,3 | *102,7* | *104,5* | | **103,1** | 10,2 | *101,9* | *104,3* | | **-0,2** | ns | **-0,5** | ns |
| 65-74 | **105,1** | 10,6 | *104,3* | *105,9* | | **104,2** | 11,0 | *103,3* | *105,2* | | **104,5** | 10,7 | *103,2* | *105,7* | | **-0,6** | ns | **0,2** | ns |

SD: standard deviation; CI: confidence interval; BMI: body mass index. ANOVA: Analysis of Variance to compare variables among age classes within periods. t-test was used to compare variables between periods. *** $p < 0.0001$; ** $p < 0.01$; *$p < 0.05$; ns: not significant p-value. 1998–2002 number of women in the 35–44, 45–54, 55–64, and 65–74 age classes (years): 711, 767, 777, and 689. 2008–2012 number of women in the 35–44, 45–54, 55–64, and 65–74 age classes (years): 504, 569, 584, and 531. 2018–2019 number of women in the 35–44, 45–54, 55–64, and 65–74 age classes (years): 232, 272, 282, and 279. Pool of the following Italian regions: Piedmont, Lombardy, Liguria, Emilia Romagna, Tuscany, Lazio, Abruzzo, Basilicata, Calabria, and Sicily.

**Table S4. Body mass index and measured height, weight, and waist and hip circumferences mean levels by educational level and period (age-adjusted using the Italian population). Italian resident men aged 35–74 years, the CUORE Project Surveys 1998–2002, 2008–2012, and 2018–2019.**

## MEN

### Body mass index (kg/m$^2$)

| Educational level | 1998-2002 | | | | ANOVA within period sign | 2008-2012 | | | | ANOVA within period sign | 2018-2019 | | | | ANOVA within period sign | 2018-2019 vs 1998-2002 | | 2018-2019 vs 2008-2012 | |
|---|---|---|---|---|---|---|---|---|---|---|---|---|---|---|---|---|---|---|---|
| | **mean** | SD | *95% CI* | | | **mean** | SD | *95% CI* | | | **mean** | SD | *95% CI* | | | **Diff** | t-test sign | **Diff** | t-test sign |
| | | | | | *** | | | | | *** | | | | | *** | | | | |
| Higher education | **26,4** | 3,7 | *26,2* | *26,6* | | **27,2** | 4,2 | *27,0* | *27,5* | | **26,7** | 4,0 | *26,4* | *27,0* | | **0,3** | ns | **-0,5** | ** |
| Lower education | **27,0** | 3,8 | *26,8* | *27,2* | | **28,0** | 4,3 | *27,7* | *28,2* | | **27,7** | 4,5 | *27,2* | *28,2* | | **0,7** | ** | **-0,3** | ns |

### Height (cm)

| Educational level | 1998-2002 | | | | ANOVA within period sign | 2008-2012 | | | | ANOVA within period sign | 2018-2019 | | | | ANOVA within period sign | 2018-2019 vs 1998-2002 | | 2018-2019 vs 2008-2012 | |
|---|---|---|---|---|---|---|---|---|---|---|---|---|---|---|---|---|---|---|---|
| | **mean** | SD | *95% CI* | | | **mean** | SD | *95% CI* | | | **mean** | SD | *95% CI* | | | **Diff** | t-test sign | **Diff** | t-test sign |
| | | | | | *** | | | | | *** | | | | | *** | | | | |
| Higher education | **172,6** | 7,1 | *172,2* | *173,0* | | **173,3** | 6,7 | *173,0* | *173,7* | | **173,9** | 6,5 | *173,4* | *174,3* | | **1,3** | *** | **0,4** | ns |
| Lower education | **170,1** | 6,9 | *169,8* | *170,4* | | **170,2** | 6,5 | *169,8* | *170,6* | | **171,3** | 7,0 | *170,5* | *172,1* | | **1,2** | ** | **1,1** | * |

### Weight (kg)

| Educational level | 1998-2002 | | | | ANOVA within period sign | 2008-2012 | | | | ANOVA within period sign | 2018-2019 | | | | ANOVA within period sign | 2018-2019 vs 1998-2002 | | 2018-2019 vs 2008-2012 | |
|---|---|---|---|---|---|---|---|---|---|---|---|---|---|---|---|---|---|---|---|
| | **mean** | SD | *95% CI* | | | **mean** | SD | *95% CI* | | | **mean** | SD | *95% CI* | | | **Diff** | t-test sign | **Diff** | t-test sign |
| Higher education | **78,6** | 12,2 | *77,9* | *79,3* | | **81,9** | 14,2 | *81,1* | *82,7* | | **80,8** | 13,1 | *79,8* | *81,7* | | **2,2** | ** | **-1,1** | ns |
| Lower education | **78,1** | 12,3 | *77,5* | *78,7* | | **81,1** | 13,4 | *80,3* | *82,0* | | **81,4** | 14,3 | *79,8* | *83,0* | | **3,3** | ** | **0,3** | ns |

### Waist to Hip circumferences ratio

| Educational level | 1998-2002 | | | | ANOVA within period sign | 2008-2012 | | | | ANOVA within period sign | 2018-2019 | | | | ANOVA within period sign | 2018-2019 vs 1998-2002 | | 2018-2019 vs 2008-2012 | |
|---|---|---|---|---|---|---|---|---|---|---|---|---|---|---|---|---|---|---|---|
| | **mean** | SD | *95% CI* | | | **mean** | SD | *95% CI* | | | **mean** | SD | *95% CI* | | | **Diff** | t-test sign | **Diff** | t-test sign |
| Higher education | **0,93** | 0,06 | *0,93* | *0,93* | | **0,95** | 0,08 | *0,95* | *0,96* | | **0,94** | 0,06 | *0,94* | *0,95* | | **0,01** | *** | **-0,01** | * |
| Lower education | **0,94** | 0,06 | *0,94* | *0,95* | | **0,97** | 0,07 | *0,96* | *0,97* | | **0,97** | 0,07 | *0,96* | *0,97* | | **0,03** | *** | **0,00** | ns |

### Waist circumferences (cm)

| Educational level | 1998-2002 | | | | ANOVA within period sign | 2008-2012 | | | | ANOVA within period sign | 2018-2019 | | | | ANOVA within period sign | 2018-2019 vs 1998-2002 | | 2018-2019 vs 2008-2012 | |
|---|---|---|---|---|---|---|---|---|---|---|---|---|---|---|---|---|---|---|---|
| | **mean** | SD | *95% CI* | | | **mean** | SD | *95% CI* | | | **mean** | SD | *95% CI* | | | **Diff** | t-test sign | **Diff** | t-test sign |
| | | | | | *** | | | | | *** | | | | | *** | | | | |
| Higher education | **94,2** | 10,4 | *93,6* | *94,8* | | **96,0** | 11,7 | *95,4* | *96,7* | | **95,2** | 10,5 | *94,5* | *96,0* | | **1,0** | * | **-0,8** | ns |
| Lower education | **95,2** | 10,6 | *94,7* | *95,7* | | **97,4** | 11,6 | *96,7* | *98,1* | | **97,7** | 11,9 | *96,3* | *99,0* | | **2,5** | ** | **0,3** | ns |

### Hip circumferences (cm)

| Educational level | 1998-2002 | | | | ANOVA within period sign | 2008-2012 | | | | ANOVA within period sign | 2018-2019 | | | | ANOVA within period sign | 2018-2019 vs 1998-2002 | | 2018-2019 vs 2008-2012 | |
|---|---|---|---|---|---|---|---|---|---|---|---|---|---|---|---|---|---|---|---|
| | **mean** | SD | *95% CI* | | | **mean** | SD | *95% CI* | | | **mean** | SD | *95% CI* | | | **Diff** | t-test sign | **Diff** | t-test sign |
| | | | | | *ns* | | | | | *ns* | | | | | *ns* | | | | |
| Higher education | **101,1** | 8,5 | *100,6* | *101,6* | | **100,9** | 9,1 | *100,3* | *101,4* | | **100,7** | 7,5 | *100,2* | *101,3* | | **-0,4** | ns | **-0,2** | ns |
| Lower education | **100,8** | 8,7 | *100,4* | *101,2* | | **100,6** | 8,3 | *100,1* | *101,1* | | **100,9** | 8,8 | *99,9* | *101,9* | | **0,1** | ns | **0,3** | ns |

Higher education—high school or university; lower education—primary or middle school. SD: standard deviation; CI: confidence interval. Means and standard deviations were age-standardized using the Italian National Institute of Statistics-ISTAT Italian population 2000, 2010, and 2019, respectively. ANOVA: Analysis of Variance to compare variables between educational levels within periods. t-test was used to compare variables between periods. *** p < 0.0001; ** p < 0.01; *p < 0.05; ns: not significant p-value. 1998–2002 number of men in the higher and lower class of education: 1249 and 1727. 2008–2012 number of men in the higher and lower class of education: 1186 and 1020. 2018–2019 number of men in the higher and lower class of education: 728 and 304. Pool of the following Italian regions: Piedmont, Lombardy, Liguria, Emilia Romagna, Tuscany, Lazio, Abruzzo, Basilicata, Calabria, and Sicily.

**Table S5. Body mass index and measured height, weight, and waist and hip circumferences mean levels by educational level and period (age-adjusted using the Italian population). Italian resident women aged 35–74 years, the CUORE Project Surveys 1998–2002, 2008–2012, and 2018–2019.**

## WOMEN

### Body mass index (kg/m$^2$)

| | 1998-2002 | | | | ANOVA within period sign | 2008-2012 | | | | ANOVA within period sign | 2018-2019 | | | | ANOVA within period sign | 2018-2019 vs 1998-2002 | | 2018-2019 vs 2008-2012 | |
|---|---|---|---|---|---|---|---|---|---|---|---|---|---|---|---|---|---|---|---|
| **Educational level** | **mean** | SD | *95% CI* | | *** | **mean** | SD | *95% CI* | | *** | **mean** | SD | *95% CI* | | *** | **Diff** | t-test sign | **Diff** | t-test sign |
| Higher education | **25,1** | 4,2 | *24,8* | *25,4* | | **25,6** | 5,0 | *25,3* | *25,9* | | **25,4** | 5,0 | *25,1* | *25,8* | | **0,3** | ns | **-0,2** | ns |
| Lower education | **27,0** | 5,1 | *26,7* | *27,2* | | **27,9** | 5,5 | *27,5* | *28,2* | | **28,3** | 5,6 | *27,7* | *28,9* | | **1,3** | *** | **0,4** | ns |

### Height (cm)

| | 1998-2002 | | | | ANOVA within period sign | 2008-2012 | | | | ANOVA within period sign | 2018-2019 | | | | ANOVA within period sign | 2018-2019 vs 1998-2002 | | 2018-2019 vs 2008-2012 | |
|---|---|---|---|---|---|---|---|---|---|---|---|---|---|---|---|---|---|---|---|
| **Educational level** | **mean** | SD | *95% CI* | | *** | **mean** | SD | *95% CI* | | *** | **mean** | SD | *95% CI* | | *** | **Diff** | t-test sign | **Diff** | t-test sign |
| Higher education | **159,6** | 6,3 | *159,2* | *159,9* | | **159,3** | 6,0 | *158,9* | *159,6* | | **159,8** | 5,8 | *159,3* | *160,2* | | **0,2** | ns | **0,5** | ns |
| Lower education | **157,3** | 6,2 | *157,0* | *157,6* | | **156,8** | 6,4 | *156,4* | *157,2* | | **157,5** | 6,4 | *156,8* | *158,2* | | **0,2** | ns | **0,7** | ns |

### Weight (kg)

| | 1998-2002 | | | | ANOVA within period sign | 2008-2012 | | | | ANOVA within period sign | 2018-2019 | | | | ANOVA within period sign | 2018-2019 vs 1998-2002 | | 2018-2019 vs 2008-2012 | |
|---|---|---|---|---|---|---|---|---|---|---|---|---|---|---|---|---|---|---|---|
| **Educational level** | **mean** | SD | *95% CI* | | *** | **mean** | SD | *95% CI* | | *** | **mean** | SD | *95% CI* | | *** | **Diff** | t-test sign | **Diff** | t-test sign |
| Higher education | **63,8** | 10,8 | *63,1* | *64,4* | | **64,9** | 12,6 | *64,1* | *65,6* | | **64,8** | 12,9 | *63,8* | *65,7* | | **1,0** | ns | **-0,1** | ns |
| Lower education | **66,6** | 12,6 | *66,0* | *67,2* | | **68,3** | 13,2 | *67,5* | *69,1* | | **70,2** | 14,2 | *68,7* | *71,8* | | **3,6** | *** | **1,9** | * |

### Waist to Hip circumferences ratio

| | 1998-2002 | | | | ANOVA within period sign | 2008-2012 | | | | ANOVA within period sign | 2018-2019 | | | | ANOVA within period sign | 2018-2019 vs 1998-2002 | | 2018-2019 vs 2008-2012 | |
|---|---|---|---|---|---|---|---|---|---|---|---|---|---|---|---|---|---|---|---|
| **Educational level** | **mean** | SD | *95% CI* | | *** | **mean** | SD | *95% CI* | | *** | **mean** | SD | *95% CI* | | *** | **Diff** | t-test sign | **Diff** | t-test sign |
| Higher education | **0,82** | 0,06 | *0,82* | *0,83* | | **0,84** | 0,08 | *0,84* | *0,85* | | **0,83** | 0,07 | *0,82* | *0,83* | | **1,00** | ns | **-0,01** | *** |
| Lower education | **0,84** | 0,06 | *0,84* | *0,85* | | **0,86** | 0,08 | *0,85* | *0,86* | | **0,85** | 0,07 | *0,85* | *0,86* | | **0,01** | * | **-0,01** | ns |

### Waist circumferences (cm)

| | 1998-2002 | | | | ANOVA within period sign | 2008-2012 | | | | ANOVA within period sign | 2018-2019 | | | | ANOVA within period sign | 2018-2019 vs 1998-2002 | | 2018-2019 vs 2008-2012 | |
|---|---|---|---|---|---|---|---|---|---|---|---|---|---|---|---|---|---|---|---|
| **Educational level** | **mean** | SD | *95% CI* | | *** | **mean** | SD | *95% CI* | | *** | **mean** | SD | *95% CI* | | *** | **Diff** | t-test sign | **Diff** | t-test sign |
| Higher education | **81,5** | 10,3 | *80,9* | *82,1* | | **84,4** | 12,0 | *83,7* | *85,1* | | **83,8** | 12,1 | *82,9* | *84,7* | | **2,3** | *** | **-0,6** | ns |
| Lower education | **85,9** | 12,0 | *85,4* | *86,4* | | **88,7** | 13,0 | *87,9* | *89,5* | | **90,3** | 13,3 | *88,9* | *91,7* | | **4,4** | *** | **1,6** | ns |

### Hip circumferences (cm)

| | 1998-2002 | | | | ANOVA within period sign | 2008-2012 | | | | ANOVA within period sign | 2018-2019 | | | | ANOVA within period sign | 2018-2019 vs 1998-2002 | | 2018-2019 vs 2008-2012 | |
|---|---|---|---|---|---|---|---|---|---|---|---|---|---|---|---|---|---|---|---|
| **Educational level** | **mean** | SD | *95% CI* | | *** | **mean** | SD | *95% CI* | | *** | **mean** | SD | *95% CI* | | *** | **Diff** | t-test sign | **Diff** | t-test sign |
| Higher education | **98,7** | 9,7 | *98,1* | *99,3* | | **100,3** | 10,5 | *99,7* | *100,9* | | **101,0** | 10,3 | *100,3* | *101,8* | | **2,3** | *** | **0,7** | ns |
| Lower education | **101,7** | 11,4 | *101,1* | *102,2* | | **103,4** | 11,1 | *102,7* | *104,1* | | **105,6** | 11,3 | *104,4* | *106,8* | | **3,9** | *** | **2,2** | ** |

Higher education—high school or university; lower education—primary or middle school. SD: standard deviation; CI: confidence interval. Means and standard deviations were age-standardized using the Italian National Institute of Statistics-ISTAT Italian population 2000, 2010, and 2019, respectively. ANOVA: Analysis of Variance to compare variables between educational levels within periods. t-test was used to compare variables between periods. *** $p < 0.0001$; ** $p < 0.01$; *$p < 0.05$; ns: not significant p-value. 1998–2002 number of women in the higher and lower class of education: 1050 and 1878. 2008–2012 number of women in the higher and lower class of education: 1153 and 1014. 2018–2019 number of women in the higher and lower class of education: 722 and 341.Pool of the following Italian regions: Piedmont, Lombardy, Liguria, Emilia Romagna, Tuscany, Lazio, Abruzzo, Basilicata, Calabria, and Sicily.

**Table S6. Prevalence of body mass index and waist and hip circumferences classes based on measurements, by age class and period. Italian resident men aged 35–74 years, the CUORE Project Surveys 1998–2002, 2008–2012, and 2018–2019.**

**MEN**

**Underweight**

| Age class (years) | 1998-2002 % | 95% CI | | Chi-squared test within period sign | 2008-2012 % | 95% CI | | Chi-squared test within period sign | 2018-2019 % | 95% CI | | Chi-squared test within period sign | 2018-2019 vs 1998-2002 Diff | chi-squared sign | 2018-2019 vs 2008-2012 Diff | chi-squared sign |
|---|---|---|---|---|---|---|---|---|---|---|---|---|---|---|---|---|
| | | | | - | | | | - | | | | - | | | | |
| 35-44 | 0,7 | 0,1 | 1,3 | | 0,6 | 0,0 | 1,2 | | 0,8 | 0,0 | 1,9 | | 0,1 | ns | 0,2 | ns |
| 45-54 | 0,5 | 0,0 | 1,0 | | 0,0 | - | - | | 0,4 | 0,0 | 1,2 | | -0,1 | ns | 0,4 | ns |
| 55-64 | 0,4 | 0,0 | 0,8 | | 0,0 | - | - | | 0,0 | - | - | | -0,4 | - | 0,0 | - |
| 65-74 | 0,8 | 0,2 | 1,5 | | 0,8 | 0,0 | 1,5 | | 0,8 | 0,0 | 1,8 | | 0,0 | ns | 0,0 | ns |

**Normal weight**

| Age class (years) | 1998-2002 % | 95% CI | | Chi-squared test within period sign | 2008-2012 % | 95% CI | | Chi-squared test within period sign | 2018-2019 % | 95% CI | | Chi-squared test within period sign | 2018-2019 vs 1998-2002 Diff | chi-squared sign | 2018-2019 vs 2008-2012 Diff | chi-squared sign |
|---|---|---|---|---|---|---|---|---|---|---|---|---|---|---|---|---|
| | | | | ** | | | | *** | | | | *** | | | | |
| 35-44 | 38 | 35 | 42 | | 37 | 33 | 41 | | 43 | 37 | 49 | | 4 | ns | 6 | ns |
| 45-54 | 32 | 29 | 36 | | 30 | 26 | 34 | | 35 | 29 | 41 | | 3 | ns | 5 | ns |
| 55-64 | 29 | 26 | 32 | | 23 | 19 | 26 | | 34 | 28 | 39 | | 5 | ns | 11 | ** |
| 65-74 | 31 | 27 | 34 | | 22 | 19 | 26 | | 22 | 17 | 27 | | -9 | ** | 0 | ns |

**Overweight**

| Age class (years) | 1998-2002 % | 95% CI | | Chi-squared test within period sign | 2008-2012 % | 95% CI | | Chi-squared test within period sign | 2018-2019 % | 95% CI | | Chi-squared test within period sign | 2018-2019 vs 1998-2002 Diff | chi-squared sign | 2018-2019 vs 2008-2012 Diff | chi-squared sign |
|---|---|---|---|---|---|---|---|---|---|---|---|---|---|---|---|---|
| | | | | ns | | | | ** | | | | * | | | | |
| 35-44 | 46 | 43 | 50 | | 43 | 39 | 48 | | 40 | 34 | 46 | | -6 | ns | -3 | ns |
| 45-54 | 50 | 46 | 53 | | 45 | 41 | 49 | | 47 | 40 | 53 | | -3 | ns | 2 | ns |
| 55-64 | 51 | 48 | 55 | | 52 | 48 | 56 | | 44 | 39 | 50 | | -7 | ns | -8 | * |
| 65-74 | 49 | 46 | 53 | | 50 | 46 | 54 | | 54 | 48 | 60 | | 5 | ns | 4 | ns |

**Obesity**

| Age class (years) | 1998-2002 % | 95% CI | | Chi-squared test within period sign | 2008-2012 % | 95% CI | | Chi-squared test within period sign | 2018-2019 % | 95% CI | | Chi-squared test within period sign | 2018-2019 vs 1998-2002 Diff | chi-squared sign | 2018-2019 vs 2008-2012 Diff | chi-squared sign |
|---|---|---|---|---|---|---|---|---|---|---|---|---|---|---|---|---|
| | | | | * | | | | * | | | | ns | | | | |
| 35-44 | 14 | 12 | 17 | | 19 | 16 | 23 | | 16 | 12 | 21 | | 2 | ns | -3 | ns |
| 45-54 | 17 | 15 | 20 | | 25 | 21 | 28 | | 18 | 13 | 22 | | 1 | ns | -7 | * |
| 55-64 | 20 | 17 | 22 | | 25 | 21 | 28 | | 22 | 17 | 27 | | 2 | ns | -3 | ns |
| 65-74 | 19 | 17 | 22 | | 27 | 23 | 31 | | 24 | 19 | 29 | | 5 | ns | -3 | ns |

**Severe obesity**

| Age class (years) | 1998-2002 % | 95% CI | | Chi-squared test within period sign | 2008-2012 % | 95% CI | | Chi-squared test within period sign | 2018-2019 % | 95% CI | | Chi-squared test within period sign | 2018-2019 vs 1998-2002 Diff | chi-squared sign | 2018-2019 vs 2008-2012 Diff | chi-squared sign |
|---|---|---|---|---|---|---|---|---|---|---|---|---|---|---|---|---|
| | | | | ns | | | | ns | | | | ns | | | | |
| 35-44 | 0,0 | - | - | | 1,5 | 0,5 | 2,5 | | 0,8 | 0,0 | 1,9 | | 0,8 | * | -0,7 | ns |
| 45-54 | 0,5 | 0,0 | 1,0 | | 1,4 | 0,4 | 2,3 | | 2,4 | 0,5 | 4,3 | | 1,9 | ** | 1,0 | ns |
| 55-64 | 0,6 | 0,1 | 1,2 | | 1,4 | 0,4 | 2,4 | | 0,7 | 0,0 | 1,7 | | 0,1 | ns | -0,7 | ns |
| 65-74 | 0,3 | 0,0 | 0,6 | | 1,1 | 0,2 | 2,0 | | 0,8 | 0,0 | 1,8 | | 0,5 | ns | -0,3 | ns |

**High waist-to-hip ratio**

| Age class (years) | 1998-2002 % | 95% CI | | Chi-squared test within period sign | 2008-2012 % | 95% CI | | Chi-squared test within period sign | 2018-2019 % | 95% CI | | Chi-squared test within period sign | 2018-2019 vs 1998-2002 Diff | chi-squared sign | 2018-2019 vs 2008-2012 Diff | chi-squared sign |
|---|---|---|---|---|---|---|---|---|---|---|---|---|---|---|---|---|
| | | | | *** | | | | *** | | | | *** | | | | |
| 35-44 | 72 | 69 | 75 | | 69 | 65 | 73 | | 63 | 57 | 69 | | -9 | ** | -6 | ns |
| 45-54 | 81 | 78 | 84 | | 83 | 79 | 86 | | 76 | 71 | 81 | | -5 | ns | -7 | * |
| 55-64 | 85 | 82 | 87 | | 89 | 87 | 92 | | 83 | 79 | 87 | | -2 | ns | -6 | * |
| 65-74 | 84 | 82 | 87 | | 92 | 89 | 94 | | 93 | 90 | 96 | | 7 | ** | 1 | ns |

**Abdominal obesity**

| Age class (years) | 1998-2002 % | 95% CI | | Chi-squared test within period sign | 2008-2012 % | 95% CI | | Chi-squared test within period sign | 2018-2019 % | 95% CI | | Chi-squared test within period sign | 2018-2019 vs 1998-2002 Diff | chi-squared sign | 2018-2019 vs 2008-2012 Diff | chi-squared sign |
|---|---|---|---|---|---|---|---|---|---|---|---|---|---|---|---|---|
| | | | | *** | | | | *** | | | | *** | | | | |
| 35-44 | 16 | 13 | 19 | | 23 | 19 | 26 | | 16 | 11 | 21 | | 0 | ns | -7 | * |
| 45-54 | 25 | 22 | 28 | | 31 | 27 | 34 | | 23 | 18 | 29 | | -2 | ns | -8 | * |
| 55-64 | 28 | 25 | 31 | | 34 | 30 | 38 | | 30 | 25 | 36 | | 2 | ns | -4 | ns |
| 65-74 | 34 | 30 | 37 | | 43 | 39 | 47 | | 38 | 33 | 44 | | 4 | ns | -5 | ns |

CI: confidence interval. Chi-squared test was used to compare prevalence between periods and among age classes within the period. Underweight: body mass index < 18.5 kg/m$^2$. Normal weight: 18.5 ≤ body mass index < 25.0 kg/m$^2$. Overweight: 25.0 ≤ body mass index < 30.0 kg/m$^2$. Obesity: body mass index ≥ 30.0 kg/m$^2$. Severe obesity: body mass index ≥ 40.0 kg/m$^2$. High waist-to-hip ratio: waist-to-hip ratio ≥ 0.90 in men and ≥ 0.85 in women. Abdominal obesity: waist circumference > 102 cm in men and > 88 cm in women. *** p < 0.0001; ** p < 0.01; *p < 0.05; ns: not significant p-value. 1998–2002 number of men in the 35–44, 45–54, 55–64, and 65–74 age classes (years): 717, 752, 784, and 731. 2008–2012 number of men in the 35–44, 45–54, 55–64, and 65–74 age classes (years): 532, 589, 567, and 536. 2018–2019 number of men in the 35–44, 45–54, 55–64, and 65–74 age classes (years): 249, 249, 277, and 260. Pool of the following Italian regions: Piedmont, Lombardy, Liguria, Emilia Romagna, Tuscany, Lazio, Abruzzo, Basilicata, Calabria, and Sicily.

**Table S7. Prevalence of body mass index and waist and hip circumferences classes based on measurements, by age class and period. Italian resident women aged 35–74 years, the CUORE Project Surveys 1998–2002, 2008–2012, and 2018–2019.**

**WOMEN**

**Underweight**

| Age class (years) | 1998-2002 | | | Chi-squared test within period sign | 2008-2012 | | | Chi-squared test within period sign | 2018-2019 | | | Chi-squared test within period sign | 2018-2019 vs 1998-2002 | | 2018-2019 vs 2008-2012 | |
|---|---|---|---|---|---|---|---|---|---|---|---|---|---|---|---|---|
| | % | 95% CI | | | % | 95% CI | | | % | 95% CI | | | Diff | chi-squared sign | Diff | chi-squared sign |
| 35-44 | 3,5 | 2,2 | 4,9 | *** | 3,0 | 1,5 | 4,5 | ** | 3,9 | 1,4 | 6,4 | ns | 0,4 | ns | 0,9 | ns |
| 45-54 | 0,9 | 0,2 | 1,6 | | 1,6 | 0,6 | 2,6 | | 2,6 | 0,7 | 4,5 | | 1,7 | * | 1,0 | ns |
| 55-64 | 2,1 | 1,1 | 3,1 | | 0,3 | 0,0 | 0,8 | | 1,4 | 0,0 | 2,8 | | -0,6 | ns | 1,1 | ns |
| 65-74 | 0,6 | 0,0 | 1,1 | | 0,9 | 0,1 | 1,8 | | 1,1 | 0,0 | 2,3 | | 0,5 | ns | 0,1 | ns |

**Normalweight**

| Age class (years) | 1998-2002 | | | Chi-squared test within period sign | 2008-2012 | | | Chi-squared test within period sign | 2018-2019 | | | Chi-squared test within period sign | 2018-2019 vs 1998-2002 | | 2018-2019 vs 2008-2012 | |
|---|---|---|---|---|---|---|---|---|---|---|---|---|---|---|---|---|
| | % | 95% CI | | | % | 95% CI | | | % | 95% CI | | | Diff | chi-squared sign | Diff | chi-squared sign |
| 35-44 | 64 | 60 | 68 | *** | 59 | 55 | 63 | *** | 60 | 54 | 67 | *** | -4 | ns | 1 | ns |
| 45-54 | 47 | 43 | 50 | | 46 | 42 | 50 | | 49 | 43 | 54 | | 2 | ns | 2 | ns |
| 55-64 | 31 | 28 | 34 | | 36 | 32 | 40 | | 43 | 37 | 49 | | 12 | ** | 7 | ns |
| 65-74 | 30 | 26 | 33 | | 25 | 22 | 29 | | 30 | 24 | 35 | | 0 | ns | 5 | ns |

**Overweight**

| Age class (years) | 1998-2002 | | | Chi-squared test within period sign | 2008-2012 | | | Chi-squared test within period sign | 2018-2019 | | | Chi-squared test within period sign | 2018-2019 vs 1998-2002 | | 2018-2019 vs 2008-2012 | |
|---|---|---|---|---|---|---|---|---|---|---|---|---|---|---|---|---|
| | % | 95% CI | | | % | 95% CI | | | % | 95% CI | | | Diff | chi-squared sign | Diff | chi-squared sign |
| 35-44 | 22 | 19 | 25 | *** | 24 | 20 | 28 | *** | 21 | 16 | 26 | ** | -1 | ns | -3 | ns |
| 45-54 | 33 | 30 | 37 | | 33 | 29 | 37 | | 25 | 20 | 30 | | -8 | ** | -8 | * |
| 55-64 | 41 | 38 | 45 | | 32 | 28 | 36 | | 32 | 26 | 37 | | -9 | ** | 0 | ns |
| 65-74 | 38 | 34 | 42 | | 41 | 37 | 45 | | 38 | 32 | 43 | | 0 | ns | -3 | ns |

**Obesity**

| Age class (years) | 1998-2002 | | | Chi-squared test within period sign | 2008-2012 | | | Chi-squared test within period sign | 2018-2019 | | | Chi-squared test within period sign | 2018-2019 vs 1998-2002 | | 2018-2019 vs 2008-2012 | |
|---|---|---|---|---|---|---|---|---|---|---|---|---|---|---|---|---|
| | % | 95% CI | | | % | 95% CI | | | % | 95% CI | | | Diff | chi-squared sign | Diff | chi-squared sign |
| 35-44 | 11 | 8 | 13 | *** | 14 | 11 | 17 | *** | 15 | 10 | 19 | ** | 4 | ns | 1 | ns |
| 45-54 | 19 | 16 | 22 | | 19 | 16 | 22 | | 24 | 19 | 29 | | 5 | ns | 5 | ns |
| 55-64 | 26 | 23 | 29 | | 31 | 27 | 35 | | 24 | 19 | 29 | | -2 | ns | -7 | * |
| 65-74 | 32 | 28 | 35 | | 33 | 29 | 37 | | 32 | 26 | 37 | | 0 | ns | -1 | ns |

**Severe obesity**

| Age class (years) | 1998-2002 | | | Chi-squared test within period sign | 2008-2012 | | | Chi-squared test within period sign | 2018-2019 | | | Chi-squared test within period sign | 2018-2019 vs 1998-2002 | | 2018-2019 vs 2008-2012 | |
|---|---|---|---|---|---|---|---|---|---|---|---|---|---|---|---|---|
| | % | 95% CI | | | % | 95% CI | | | % | 95% CI | | | Diff | chi-squared sign | Diff | chi-squared sign |
| 35-44 | 1,3 | 0,4 | 2,1 | ns | 1,6 | 0,5 | 2,7 | ns | 1,3 | 0,0 | 2,7 | ns | 0,0 | ns | -0,3 | ns |
| 45-54 | 2,1 | 1,1 | 3,1 | | 2,8 | 1,5 | 4,2 | | 3,7 | 1,4 | 5,9 | | 1,6 | ns | 0,9 | ns |
| 55-64 | 2,3 | 1,3 | 3,4 | | 2,2 | 1,0 | 3,4 | | 1,1 | 0,0 | 2,3 | | -1,2 | ns | -1,1 | ns |
| 65-74 | 1,7 | 0,8 | 2,7 | | 2,3 | 1,0 | 3,5 | | 2,2 | 0,4 | 3,9 | | 0,5 | ns | -0,1 | ns |

**High waist-to-hip ratio**

| Age class (years) | 1998-2002 | | | Chi-squared test within period sign | 2008-2012 | | | Chi-squared test within period sign | 2018-2019 | | | Chi-squared test within period sign | 2018-2019 vs 1998-2002 | | 2018-2019 vs 2008-2012 | |
|---|---|---|---|---|---|---|---|---|---|---|---|---|---|---|---|---|
| | % | 95% CI | | | % | 95% CI | | | % | 95% CI | | | Diff | chi-squared sign | Diff | chi-squared sign |
| 35-44 | 30 | 26 | 33 | *** | 38 | 34 | 42 | *** | 22 | 17 | 27 | *** | -8 | * | -16 | *** |
| 45-54 | 38 | 34 | 41 | | 45 | 41 | 49 | | 34 | 29 | 40 | | -4 | ns | -11 | ** |
| 55-64 | 52 | 48 | 56 | | 55 | 51 | 59 | | 45 | 40 | 51 | | -7 | ns | -10 | ** |
| 65-74 | 61 | 58 | 65 | | 66 | 62 | 70 | | 67 | 62 | 73 | | 6 | ns | 1 | ns |

**Abdominal obesity**

| Age class (years) | 1998-2002 | | | Chi-squared test within period sign | 2008-2012 | | | Chi-squared test within period sign | 2018-2019 | | | Chi-squared test within period sign | 2018-2019 vs 1998-2002 | | 2018-2019 vs 2008-2012 | |
|---|---|---|---|---|---|---|---|---|---|---|---|---|---|---|---|---|
| | % | 95% CI | | | % | 95% CI | | | % | 95% CI | | | Diff | chi-squared sign | Diff | chi-squared sign |
| 35-44 | 16 | 14 | 19 | *** | 28 | 24 | 32 | *** | 21 | 15 | 26 | *** | 5 | ns | -7 | * |
| 45-54 | 29 | 26 | 32 | | 38 | 34 | 42 | | 34 | 29 | 40 | | 5 | ns | -4 | ns |
| 55-64 | 46 | 43 | 50 | | 51 | 46 | 55 | | 47 | 41 | 53 | | 1 | ns | -4 | ns |
| 65-74 | 59 | 55 | 62 | | 63 | 59 | 67 | | 62 | 57 | 68 | | 3 | ns | -1 | ns |

CI: confidence interval. Chi-squared test was used to compare prevalence between periods and among age classes within the period. Underweight: body mass index < 18.5 kg/m$^2$. Normal weight: 18.5 ≤ body mass index < 25.0 kg/m$^2$. Overweight: 25.0 ≤ body mass index < 30.0 kg/m$^2$. Obesity: body mass index ≥ 30.0 kg/m$^2$. Severe obesity: body mass index ≥ 40.0 kg/m$^2$. High waist-to-hip ratio: waist-to-hip ratio ≥ 0.90 in men and ≥ 0.85 in women. Abdominal obesity: waist circumference > 102 cm in men and > 88 cm in women. *** p < 0.0001; ** p < 0.01; *p < 0.05; ns: not significant p-value. 1998–2002 number of women in the 35–44, 45–54, 55–64, and 65–74 age classes (years): 711, 767, 777, and 689. 2008–2012 number of women in the 35–44, 45–54, 55–64, and 65–74 age classes (years): 504, 569, 584, and 531. 2018–2019 number of women in the 35–44, 45–54, 55–64, and 65–74 age classes (years): 232, 272, 282, and 279. Pool of the following Italian regions: Piedmont, Lombardy, Liguria, Emilia Romagna, Tuscany, Lazio, Abruzzo, Basilicata, Calabria, and Sicily.

**Table S8. Prevalence of body mass index and waist and hip circumferences classes based on measurements, by educational level and period (age-adjusted using the Italian population). Italian resident men aged 35–74 years, the CUORE Project Surveys 1998–2002, 2008–2012, and 2018–2019.**

| MEN | | | | | | | | | | | | | | | | |
|---|---|---|---|---|---|---|---|---|---|---|---|---|---|---|---|---|
| **Normal weight** | | | | | | | | | | | | | | | | |
| | **1998-2002** | | | Chi-squared test within period | **2008-2012** | | | Chi-squared test within period | **2018-2019** | | | Chi-squared test within period | **2018-2019 vs 1998-2002** | | **2018-2019 vs 2008-2012** | |
| **Educational level** | **%** | *95% CI* | | *sign* | **%** | *95% CI* | | *sign* | **%** | *95% CI* | | *sign* | **Diff** | chi-squared *sign* | **Diff** | chi-squared *sign* |
| Higher education | **37** | *34* | *39* | ** | **31** | *28* | *33* | * | **36** | *33* | *40* | ** | **-1** | ns | **5** | ** |
| Lower education | **30** | *28* | *33* | | **26** | *24* | *29* | | **28** | *23* | *33* | | **-2** | ns | **2** | ns |
| **Overweight** | | | | | | | | | | | | | | | | |
| | **1998-2002** | | | Chi-squared test within period | **2008-2012** | | | Chi-squared test within period | **2018-2019** | | | Chi-squared test within period | **2018-2019 vs 1998-2002** | | **2018-2019 vs 2008-2012** | |
| **Educational level** | **%** | *95% CI* | | *sign* | **%** | *95% CI* | | *sign* | **%** | *95% CI* | | *sign* | **Diff** | chi-squared *sign* | **Diff** | chi-squared *sign* |
| Higher education | **49** | *46* | *51* | *ns* | **48** | *45* | *51* | *ns* | **46** | *42* | *49* | *ns* | **-3** | ns | **-2** | ns |
| Lower education | **50** | *47* | *52* | | **46** | *43* | *49* | | **47** | *41* | *52* | | **-3** | ns | **1** | ns |
| **Obesity** | | | | | | | | | | | | | | | | |
| | **1998-2002** | | | Chi-squared test within period | **2008-2012** | | | Chi-squared test within period | **2018-2019** | | | Chi-squared test within period | **2018-2019 vs 1998-2002** | | **2018-2019 vs 2008-2012** | |
| **Educational level** | **%** | *95% CI* | | *sign* | **%** | *95% CI* | | *sign* | **%** | *95% CI* | | *sign* | **Diff** | chi-squared *sign* | **Diff** | chi-squared *sign* |
| Higher education | **14** | *12* | *16* | ** | **21** | *19* | *24* | ** | **17** | *15* | *20* | ** | **3** | ns | **-4** | * |
| Lower education | **19** | *18* | *21* | | **27** | *25* | *30* | | **25** | *20* | *30* | | **6** | * | **-2** | ns |
| **High waist-to-hip ratio** | | | | | | | | | | | | | | | | |
| | **1998-2002** | | | Chi-squared test within period | **2008-2012** | | | Chi-squared test within period | **2018-2019** | | | Chi-squared test within period | **2018-2019 vs 1998-2002** | | **2018-2019 vs 2008-2012** | |
| **Educational level** | **%** | *95% CI* | | *sign* | **%** | *95% CI* | | *sign* | **%** | *95% CI* | | *sign* | **Diff** | chi-squared *sign* | **Diff** | chi-squared *sign* |
| Higher education | **77** | *75* | *80* | ** | **79** | *77* | *82* | ** | **76** | *73* | *79* | ** | **-1** | ns | **-3** | ns |
| Lower education | **82** | *81* | *84* | | **85** | *83* | *88* | | **84** | *80* | *88* | | **2** | ns | **-1** | ns |
| **Abdominal obesity** | | | | | | | | | | | | | | | | |
| | **1998-2002** | | | Chi-squared test within period | **2008-2012** | | | Chi-squared test within period | **2018-2019** | | | Chi-squared test within period | **2018-2019 vs 1998-2002** | | **2018-2019 vs 2008-2012** | |
| **Educational level** | **%** | *95% CI* | | *sign* | **%** | *95% CI* | | *sign* | **%** | *95% CI* | | *sign* | **Diff** | chi-squared *sign* | **Diff** | chi-squared *sign* |
| Higher education | **21** | *19* | *24* | ** | **29** | *26* | *32* | ** | **23** | *20* | *26* | ** | **2** | ns | **-6** | ** |
| Lower education | **26** | *24* | *28* | | **34** | *31* | *37* | | **32** | *27* | *38* | | **6** | * | **-2** | ns |

Higher education—high school or university; lower education—primary or middle school. Prevalence were age-standardized using the Italian National Institute of Statistics-ISTAT Italian population 2000, 2010, and 2019, respectively. Chi-squared test was used to compare prevalence between periods and among educational levels within the period. Normal weight: 18.5 ≤ body mass index < 25.0 kg/m$^2$. Overweight: 25.0 ≤ body mass index < 30.0 kg/m$^2$. Obesity: body mass index ≥ 30.0 kg/m$^2$. High waist-to-hip ratio: waist-to-hip ratio ≥ 0.90 in men and ≥ 0.85 in women. Abdominal obesity: waist circumference > 102 cm in men and > 88 cm in women. *** p < 0.0001; ** p < 0.01; *p < 0.05; ns: not significant p-value. 1998–2002 number of men in the higher and lower class of education: 1249 and 1727. 2008–2012 number of men in the higher and lower class of education: 1186 and 1020. 2018–2019 number of men in the higher and lower class of education: 728 and 304. Pool of the following Italian regions: Piedmont, Lombardy, Liguria, Emilia Romagna, Tuscany, Lazio, Abruzzo, Basilicata, Calabria, and Sicily.

**Table S9. Prevalence of body mass index and waist and hip circumferences classes based on measurements, by educational level and period (age-adjusted using the Italian population). Italian resident women aged 35–74 years, the CUORE Project Surveys 1998–2002, 2008–2012, and 2018–2019.**

**WOMEN**

**Normal weight**

| Educational level | 1998-2002 % | 95% CI | | Chi-squared test within period sign | 2008-2012 % | 95% CI | | Chi-squared test within period sign | 2018-2019 % | 95% CI | | Chi-squared test within period sign | 2018-2019 vs 1998-2002 Diff | chi-squared sign | 2018-2019 vs 2008-2012 Diff | chi-squared sign |
|---|---|---|---|---|---|---|---|---|---|---|---|---|---|---|---|---|
| Higher education | **55** | *52* | *58* | *** | **51** | *48* | *54* | *** | **54** | *50* | *58* | *** | **-1** | ns | **3** | ns |
| Lower education | **38** | *36* | *40* | | **34** | *31* | *37* | | **27** | *22* | *31* | | **-11** | ** | **-7** | * |

**Overweight**

| Educational level | 1998-2002 % | 95% CI | | Chi-squared test within period sign | 2008-2012 % | 95% CI | | Chi-squared test within period sign | 2018-2019 % | 95% CI | | Chi-squared test within period sign | 2018-2019 vs 1998-2002 Diff | chi-squared sign | 2018-2019 vs 2008-2012 Diff | chi-squared sign |
|---|---|---|---|---|---|---|---|---|---|---|---|---|---|---|---|---|
| Higher education | **29** | *27* | *32* | ** | **32** | *29* | *34* | *ns* | **25** | *22* | *28* | *** | **-4** | * | **-7** | ** |
| Lower education | **36** | *33* | *38* | | **34** | *31* | *37* | | **38** | *33* | *43* | | **2** | ns | **4** | ns |

**Obesity**

| Educational level | 1998-2002 % | 95% CI | | Chi-squared test within period sign | 2008-2012 % | 95% CI | | Chi-squared test within period sign | 2018-2019 % | 95% CI | | Chi-squared test within period sign | 2018-2019 vs 1998-2002 Diff | chi-squared sign | 2018-2019 vs 2008-2012 Diff | chi-squared sign |
|---|---|---|---|---|---|---|---|---|---|---|---|---|---|---|---|---|
| Higher education | **14** | *12* | *16* | *** | **15** | *13* | *18* | *** | **19** | *16* | *22* | *** | **5** | ** | **4** | * |
| Lower education | **25** | *23* | *27* | | **31** | *28* | *34* | | **33** | *28* | *38* | | **8** | ** | **2** | ns |

**High waist-to-hip ratio**

| Educational level | 1998-2002 % | 95% CI | | Chi-squared test within period sign | 2008-2012 % | 95% CI | | Chi-squared test within period sign | 2018-2019 % | 95% CI | | Chi-squared test within period sign | 2018-2019 vs 1998-2002 Diff | chi-squared sign | 2018-2019 vs 2008-2012 Diff | chi-squared sign |
|---|---|---|---|---|---|---|---|---|---|---|---|---|---|---|---|---|
| Higher education | **35** | *32* | *38* | *** | **44** | *41* | *47* | *** | **36** | *33* | *40* | *** | **1** | ns | **-8** | ** |
| Lower education | **49** | *46* | *51* | | **56** | *53* | *59* | | **52** | *47* | *57* | | **3** | ns | **-4** | ns |

**Abdominal obesity**

| Educational level | 1998-2002 % | 95% CI | | Chi-squared test within period sign | 2008-2012 % | 95% CI | | Chi-squared test within period sign | 2018-2019 % | 95% CI | | Chi-squared test within period sign | 2018-2019 vs 1998-2002 Diff | chi-squared sign | 2018-2019 vs 2008-2012 Diff | chi-squared sign |
|---|---|---|---|---|---|---|---|---|---|---|---|---|---|---|---|---|
| Higher education | **26** | *23* | *29* | *** | **35** | *33* | *38* | *** | **34** | *31* | *37* | *** | **8** | ** | **-1** | ns |
| Lower education | **41** | *39* | *43* | | **53** | *49* | *56* | | **54** | *49* | *59* | | **13** | *** | **1** | ns |

Higher education—high school or university; lower education—primary or middle school. Chi-squared test was used to compare prevalence between periods and among educational levels within the period. Normal weight: 18.5 ≤ body mass index < 25.0 kg/m$^2$. Overweight: 25.0 ≤ body mass index < 30.0 kg/m$^2$. Obesity: body mass index ≥ 30.0 kg/m$^2$. High waist-to-hip ratio: waist-to-hip ratio ≥ 0.90 in men and ≥ 0.85 in women. Abdominal obesity: waist circumference > 102 cm in men and > 88 cm in women. *** p < 0.0001; ** p < 0.01; *p < 0.05; ns: not significant p-value. 1998-2002 number of women in the higher and lower class of education: 1050 and 1878. 2008–2012 number of women in the higher and lower class of education: 1153 and 1014. 2018–2019 number of women in the higher and lower class of education: 722 and 341. Prevalence were age-standardized using the Italian National Institute of Statistics-ISTAT Italian population 2000, 2010, and 2019, respectively. Pool of the following Italian regions: Piedmont, Lombardy, Liguria, Emilia Romagna, Tuscany, Lazio, Abruzzo, Basilicata, Calabria, and Sicily.

**Table S10. Body mass index, normal weight, overweight and obesity based on measurements, by sex and Italian region (age-adjusted using the Italian population). Italian resident men and women aged 35–74 years, the CUORE Project Survey 2018–2019.**

**MEN**

| Italian Region | n | Body mass index (kg/m²) mean | SD | 95%CI | | Normal weight % | 95% CI | | Overweight % | 95% CI | | Obesity % | 95% CI | |
|---|---|---|---|---|---|---|---|---|---|---|---|---|---|---|
| Abruzzo | 105 | **28,3** | 3,8 | *27,6* | *29,1* | **23,3** | *15,2* | *31,3* | **49,8** | *40,3* | *59,4* | **26,9** | *18,4* | *35,4* |
| Calabria | 102 | **28,5** | 3,7 | *27,7* | *29,2* | **15,7** | *8,6* | *22,8* | **56,6** | *47,0* | *66,2* | **27,7** | *19,0* | *36,4* |
| Liguria | 104 | **26,6** | 3,4 | *25,9* | *27,2* | **36,0** | *26,8* | *45,2* | **44,1** | *34,6* | *53,6* | **19,0** | *11,4* | *26,5* |
| Lazio | 99 | **26,7** | 3,3 | *26,1* | *27,4* | **31,2** | *22,1* | *40,3* | **50,1** | *40,3* | *60,0* | **17,5** | *10,0* | *25,0* |
| Lombardy | 98 | **25,4** | 3,7 | *24,7* | *26,2* | **48,1** | *38,2* | *58,0* | **37,0** | *27,4* | *46,6* | **13,6** | *6,8* | *20,3* |
| Piedmont | 104 | **25,7** | 3,5 | *25,0* | *26,4* | **45,3** | *35,8* | *54,9* | **42,1** | *32,6* | *51,6* | **11,3** | *5,2* | *17,4* |
| Emilia Romagna | 104 | **27,3** | 4,1 | *26,5* | *28,1* | **32,6** | *23,6* | *41,6* | **46,4** | *36,8* | *56,0* | **21,0** | *13,2* | *28,8* |
| Basilicata | 106 | **27,9** | 4,1 | *27,2* | *28,7* | **31,5** | *22,6* | *40,3* | **44,0** | *34,6* | *53,5* | **24,5** | *16,3* | *32,7* |
| Tuscany | 108 | **25,6** | 3,1 | *25,0* | *26,2* | **48,7** | *39,2* | *58,1* | **42,3** | *33,0* | *51,6* | **8,2** | *3,0* | *13,4* |
| Sicily | 105 | **28,2** | 5,2 | *27,2* | *29,2* | **29,3** | *20,6* | *38,0* | **42,0** | *32,6* | *51,4* | **28,7** | *20,0* | *37,3* |

**WOMEN**

| Italian Region | n | Body mass index (kg/m²) mean | SD | 95%CI | | Normal weight % | 95% CI | | Overweight % | 95% CI | | Obesity % | 95% CI | |
|---|---|---|---|---|---|---|---|---|---|---|---|---|---|---|
| Abruzzo | 106 | **26,0** | 5,0 | *25,0* | *26,9* | **54,2** | *44,7* | *63,7* | **22,2** | *14,3* | *30,1* | **22,5** | *14,6* | *30,5* |
| Calabria | 108 | **28,8** | 6,2 | *27,6* | *29,9* | **26,1** | *17,9* | *34,4* | **36,9** | *27,8* | *46,0* | **35,2** | *26,1* | *44,2* |
| Liguria | 107 | **24,4** | 4,3 | *23,6* | *25,3* | **59,9** | *50,6* | *69,2* | **27,2** | *18,8* | *35,6* | **9,8** | *4,2* | *15,4* |
| Lazio | 113 | **25,8** | 4,5 | *25,0* | *26,6* | **51,2** | *42,0* | *60,5* | **29,3** | *20,9* | *37,7* | **18,3** | *11,1* | *25,4* |
| Lombardy | 100 | **25,4** | 4,6 | *24,4* | *26,3* | **49,5** | *39,7* | *59,3* | **28,3** | *19,4* | *37,1* | **18,5** | *10,9* | *26,1* |
| Piedmont | 113 | **23,8** | 4,0 | *23,1* | *24,6* | **64,0** | *55,1* | *72,8* | **20,2** | *12,8* | *27,7* | **12,3** | *6,2* | *18,4* |
| Emilia Romagna | 105 | **28,3** | 5,7 | *27,2* | *29,4* | **27,6** | *19,1* | *36,2* | **32,5** | *23,5* | *41,5* | **37,0** | *27,7* | *46,2* |
| Basilicata | 112 | **28,1** | 5,2 | *27,1* | *29,0* | **30,7** | *22,2* | *39,3* | **28,6** | *20,2* | *36,9* | **37,9** | *29,0* | *46,9* |
| Tuscany | 98 | **25,2** | 4,6 | *24,3* | *26,1* | **56,2** | *46,4* | *66,0* | **25,1** | *16,5* | *33,7* | **17,7** | *10,2* | *25,3* |
| Sicily | 103 | **26,7** | 4,4 | *25,9* | *27,6* | **39,8** | *30,4* | *49,3* | **33,8** | *24,6* | *42,9* | **24,6** | *16,3* | *32,9* |

Means, standard deviations, and prevalence were age-standardized using the Italian National Institute of Statistics-ISTAT Italian population 2019. SD: standard deviation; CI: confidence interval. Normal weight: 18.5 ≤ body mass index < 25.0 kg/m². Overweight: 25.0 ≤ body mass index < 30.0 kg/m². Obesity: body mass index ≥ 30.0 kg/m². Regional data for 1998–2002 and 2008–2012. CUORE Project surveys were available at http://www.cuore.iss.it/eng/survey/cuoredata.

**Table S11. Height, weight, waist and hip circumferences, and waist to hip ratio based on measurements, by sex and Italian region (age-adjusted using the Italian population). Italian resident men and women aged 35–74 years, the CUORE Project Survey 2018–2019.**

**MEN**

| | | Heignt (cm) | | | | Weight (kg) | | | | Waist circumference (cm) | | | | Hip circumference (cm) | | | | Waist to hip ratio | | | |
|---|---|---|---|---|---|---|---|---|---|---|---|---|---|---|---|---|---|---|---|---|---|
| **Italian Region** | n | **mean** | SD | *95%CI* | | **mean** | SD | *95%CI* | | **mean** | SD | *95%CI* | | **mean** | SD | *95%CI* | | **mean** | SD | *95%CI* | |
| **Abruzzo** | 105 | **172,9** | 5,8 | *171,8* | *174,1* | **84,7** | 12,1 | *82,4* | *87,1* | **100,3** | 9,6 | *98,5* | *102,2* | **102,4** | 7,3 | *101,0* | *103,8* | **0,98** | 0,06 | *0,97* | *0,99* |
| **Calabria** | 102 | **171,3** | 6,2 | *170,1* | *172,5* | **83,6** | 12,0 | *81,2* | *85,9* | **98,0** | 9,3 | *96,2* | *99,8* | **101,3** | 7,4 | *99,9* | *102,8* | **0,97** | 0,05 | *0,96* | *0,98* |
| **Liguria** | 104 | **173,7** | 6,0 | *172,5* | *174,8* | **80,4** | 12,3 | *78,0* | *82,7* | **94,6** | 9,6 | *92,8* | *96,5* | **100,5** | 6,9 | *99,2* | *101,9* | **0,94** | 0,05 | *0,93* | *0,95* |
| **Lazio** | 99 | **172,0** | 6,7 | *170,7* | *173,4* | **79,1** | 11,2 | *76,9* | *81,3* | **95,6** | 9,3 | *93,8* | *97,4* | **100,8** | 6,5 | *99,5* | *102,1* | **0,95** | 0,06 | *0,94* | *0,96* |
| **Lombardy** | 98 | **173,5** | 6,0 | *172,3* | *174,7* | **76,6** | 12,6 | *74,1* | *79,1* | **92,3** | 10,6 | *90,2* | *94,4* | **97,8** | 6,9 | *96,5* | *99,2* | **0,94** | 0,06 | *0,93* | *0,95* |
| **Piedmont** | 104 | **176,1** | 6,6 | *174,9* | *177,4* | **79,6** | 11,2 | *77,5* | *81,8* | **92,4** | 9,9 | *90,5* | *94,3* | **99,9** | 6,0 | *98,7* | *101,0* | **0,92** | 0,07 | *0,91* | *0,94* |
| **Emilia Romagna** | 104 | **174,9** | 6,7 | *173,6* | *176,2* | **83,6** | 14,5 | *80,8* | *86,4* | **95,9** | 10,8 | *93,9* | *98,0* | **102,2** | 8,7 | *100,5* | *103,9* | **0,94** | 0,06 | *0,93* | *0,95* |
| **Basilicata** | 106 | **171,2** | 6,2 | *170,0* | *172,4* | **82,1** | 13,5 | *79,5* | *84,6* | **98,9** | 10,3 | *96,9* | *100,9* | **101,7** | 7,4 | *100,3* | *103,2* | **0,97** | 0,06 | *0,96* | *0,98* |
| **Tuscany** | 108 | **175,3** | 6,1 | *174,1* | *176,4* | **78,6** | 10,4 | *76,7* | *80,6* | **93,7** | 8,9 | *92,0* | *95,3* | **100,0** | 6,4 | *98,8* | *101,3* | **0,94** | 0,06 | *0,92* | *0,95* |
| **Sicily** | 105 | **170,6** | 6,5 | *169,4* | *171,9* | **82,1** | 15,8 | *79,1* | *85,1* | **98,0** | 13,4 | *95,5* | *100,6* | **101,4** | 10,2 | *99,4* | *103,3* | **0,97** | 0,06 | *0,95* | *0,98* |

**WOMEN**

| | | Heignt (cm) | | | | Weight (kg) | | | | Waist circumference (cm) | | | | Hip circumference (cm) | | | | Waist to hip ratio | | | |
|---|---|---|---|---|---|---|---|---|---|---|---|---|---|---|---|---|---|---|---|---|---|
| **Italian Region** | n | **mean** | SD | *95%CI* | | **mean** | SD | *95%CI* | | **mean** | SD | *95%CI* | | **mean** | SD | *95%CI* | | **mean** | SD | *95%CI* | |
| **Abruzzo** | 106 | **158,8** | 5,0 | *157,8* | *159,7* | **65,4** | 12,5 | *63,0* | *67,8* | **85,8** | 13,0 | *83,3* | *88,3* | **101,1** | 10,3 | *99,1* | *103,1* | **0,85** | 0,06 | *0,83* | *0,86* |
| **Calabria** | 108 | **157,6** | 5,7 | *156,5* | *158,7* | **71,4** | 16,1 | *68,4* | *74,4* | **90,9** | 14,3 | *88,2* | *93,6* | **105,4** | 11,5 | *103,3* | *107,6* | **0,86** | 0,07 | *0,85* | *0,87* |
| **Liguria** | 107 | **160,7** | 5,8 | *159,6* | *161,8* | **62,9** | 10,1 | *61,0* | *64,8* | **81,5** | 10,1 | *79,6* | *83,4* | **99,5** | 8,8 | *97,8* | *101,2* | **0,82** | 0,05 | *0,81* | *0,83* |
| **Lazio** | 113 | **160,0** | 5,3 | *159,0* | *161,0* | **65,9** | 11,5 | *63,8* | *68,0* | **86,6** | 10,8 | *84,6* | *88,6* | **102,9** | 9,5 | *101,1* | *104,6* | **0,84** | 0,06 | *0,83* | *0,85* |
| **Lombardy** | 100 | **159,6** | 6,0 | *158,4* | *160,8* | **64,4** | 11,2 | *62,2* | *66,6* | **83,9** | 12,1 | *81,5* | *86,3* | **100,3** | 9,9 | *98,4* | *102,3* | **0,83** | 0,07 | *0,82* | *0,85* |
| **Piedmont** | 113 | **161,2** | 6,0 | *160,1* | *162,3* | **61,8** | 10,3 | *59,9* | *63,7* | **78,4** | 9,9 | *76,5* | *80,2* | **99,1** | 8,3 | *97,6* | *100,6* | **0,79** | 0,06 | *0,78* | *0,80* |
| **Emilia Romagna** | 105 | **159,1** | 5,6 | *158,0* | *160,2* | **71,6** | 15,4 | *68,7* | *74,6* | **87,9** | 13,0 | *85,4* | *90,4* | **106,0** | 11,6 | *103,7* | *108,2* | **0,83** | 0,06 | *0,82* | *0,84* |
| **Basilicata** | 112 | **157,3** | 5,8 | *156,2* | *158,4* | **69,4** | 13,8 | *66,8* | *71,9* | **91,5** | 12,4 | *89,3* | *93,8* | **105,7** | 11,1 | *103,6* | *107,7* | **0,87** | 0,06 | *0,85* | *0,88* |
| **Tuscany** | 98 | **159,5** | 5,3 | *158,5* | *160,6* | **64,1** | 11,7 | *61,8* | *66,5* | **85,9** | 12,2 | *83,5* | *88,3* | **100,9** | 9,7 | *99,0* | *102,8* | **0,85** | 0,07 | *0,84* | *0,86* |
| **Sicily** | 103 | **156,5** | 6,8 | *155,2* | *157,8* | **65,5** | 12,1 | *63,1* | *67,8* | **84,3** | 10,1 | *82,3* | *86,2* | **101,6** | 9,6 | *99,7* | *103,4* | **0,83** | 0,05 | *0,82* | *0,84* |

Means, standard deviations, and prevalence were age-standardized using the Italian National Institute of Statistics-ISTAT Italian population 2019. SD: standard deviation; CI: confidence interval. Regional data for 1998–2002 and 2008–2012. CUORE Project surveys were available at http://www.cuore.iss.it/eng/survey/cuoredata.

**Table S12. Anthropometric measurements by sex and period (age-adjusted using the European standard population). Italian resident men and women aged 35–74 years, the CUORE Project Surveys 1998–2002, 2008–2012, and 2018–2019.**

**MEN**

| | 1998-2002 *n=2984* | | | | 2008-2012 *n=2224* | | | | 2018-2019 *n=1035* | | | | 2018-2019 vs 1998-2002 | | 2018-2019 vs 2008-2012 | |
|---|---|---|---|---|---|---|---|---|---|---|---|---|---|---|---|---|
| | **mean** | SD | *95% CI* | | **mean** | SD | *95% CI* | | **mean** | SD | *95% CI* | | **Diff** | t-test p-value | **Diff** | t-test p-value |
| **BMI (kg/m$^2$)** | **26,8** | 3,8 | *26,6* | *26,9* | **27,6** | 4,3 | *27,4* | *27,7* | **27,0** | 4,2 | *26,8* | *27,3* | **0,2** | ns | **-0,6** | ** |
| **Height (cm)** | **171,0** | 7,1 | *170,7* | *171,2* | **171,8** | 6,8 | *171,5* | *172,0* | **173,2** | 6,8 | *172,7* | *173,6* | **2,2** | *** | **1,4** | *** |
| **Weight (kg)** | **78,2** | 12,3 | *77,8* | *78,7* | **81,3** | 13,8 | *80,8* | *81,9* | **81,1** | 13,5 | *80,2* | *81,9* | **2,9** | *** | **-0,2** | ns |
| **Waist/Hip** | **0,94** | 0,06 | *0,94* | *0,94* | **0,96** | 0,07 | *0,96* | *0,96* | **0,95** | 0,06 | *0,95* | *0,95* | **0,01** | *** | **-0,01** | ** |
| **Waist (cm)** | **95,0** | 10,6 | *94,6* | *95,4* | **96,8** | 11,7 | *96,3* | *97,2* | **96,0** | 11,0 | *95,3* | *96,7* | **1,0** | ** | **-0,8** | ns |
| **Hip (cm)** | **101,1** | 8,7 | *100,8* | *101,4* | **100,7** | 8,7 | *100,4* | *101,1* | **100,9** | 7,9 | *100,4* | *101,3* | **-0,2** | ns | **0,2** | ns |
| | **%** | *95% CI* | | | **%** | *95% CI* | | | **%** | *95% CI* | | | **Diff** | chi-squared p-value | **Diff** | chi-squared p-value |
| **Normalweight** | **33** | *31* | *35* | | **28** | *27* | *30* | | **34** | *31* | *37* | | **1** | ns | **6** | *** |
| **Overweight** | **49** | *47* | *51* | | **47** | *45* | *50* | | **46** | *43* | *49* | | **-3** | ns | **-1** | *** |
| **Obese** | **18** | *16* | *19* | | **24** | *22* | *26* | | **23** | *21* | *26* | | **5** | *** | **-1** | *** |
| **High waist-to-hip ratio** | **80** | *79* | *82* | | **82** | *81* | *84* | | **77** | *75* | *80* | | **-3** | ns | **-5** | *** |
| **Abdominal obesity** | **25** | *23* | *27* | | **32** | *30* | *34* | | **26** | *23* | *29* | | **1** | ns | **-6** | *** |

**WOMEN**

| | 1998-2002 ***n=2944*** | | | | 2008-2012 ***n=2188*** | | | | 2018-2019 ***n=1065*** | | | | 2018-2019 vs 1998-2002 | | 2018-2019 vs 2008-2012 | |
|---|---|---|---|---|---|---|---|---|---|---|---|---|---|---|---|---|
| | **mean** | SD | *95% CI* | | **mean** | SD | *95% CI* | | **mean** | SD | *95% CI* | | **Diff** | t-test p-value | **Diff** | t-test p-value |
| **BMI (kg/m$^2$)** | **26,3** | 4,9 | *26,1* | *26,4* | **26,7** | 5,3 | *26,5* | *26,9* | **26,2** | 5,3 | *25,9* | *26,5* | **-0,1** | | **-0,5** | |
| **Height (cm)** | **158,1** | 6,3 | *157,8* | *158,3* | **158,1** | 6,3 | *157,8* | *158,3* | **159,1** | 6,1 | *158,7* | *159,5* | **1,0** | *** | **1,0** | *** |
| **Weight (kg)** | **65,4** | 12,1 | *65,0* | *65,9* | **66,5** | 13,1 | *66,0* | *67,0* | **66,2** | 13,6 | *65,4* | *67,0* | **0,8** | ns | **-0,3** | ns |
| **Waist/Hip** | **0,84** | 0,06 | *0,83* | *0,84* | **0,85** | 0,08 | *0,85* | *0,85* | **0,83** | 0,07 | *0,83* | *0,84* | **-0,01** | ns | **-0,02** | *** |
| **Waist (cm)** | **84,2** | 11,6 | *83,8* | *84,6* | **86,6** | 12,7 | *86,1* | *87,2* | **85,5** | 12,9 | *84,7* | *86,3* | **1,3** | ** | **-1,1** | * |
| **Hip (cm)** | **100,5** | 10,9 | *100,1* | *100,9* | **101,8** | 10,9 | *101,4* | *102,3* | **102,2** | 10,8 | *101,6* | *102,9* | **1,7** | *** | **0,4** | ns |
| | **%** | *95% CI* | | | **%** | *95% CI* | | | **%** | *95% CI* | | | **Diff** | chi-squared p-value | **Diff** | chi-squared p-value |
| **Normalweight** | **44** | *42* | *46* | | **43** | *41* | *45* | | **47** | *44* | *50* | | **3** | | **4** | |
| **Overweight** | **33** | *31* | *35* | | **32** | *30* | *34* | | **28** | *25* | *31* | | **-5** | ** | **-4** | *** |
| **Obese** | **21** | *19* | *22* | | **23** | *22* | *25* | | **20** | *17* | *22* | | **-1** | ns | **-3** | *** |
| **High waist-to-hip ratio** | **44** | *42* | *46* | | **50** | *48* | *52* | | **40** | *37* | *43* | | **-4** | ns | **-10** | *** |
| **Abdominal obesity** | **36** | *34* | *38* | | **44** | *42* | *46* | | **39** | *36* | *42* | | **3** | * | **-5** | *** |

SD: standard deviation; CI: confidence interval; BMI: body mass index. Means, standard deviations and prevalence were age-standardized using the European Standard Population 2013. t-test was used to compare variables between periods; chi-squared test was used to compare prevalence between periods. Normal weight: 18.5 ≤ body mass index < 25.0 kg/m$^2$. Overweight: 25.0 ≤ body mass index < 30.0 kg/m$^2$. Obesity: body mass index ≥ 30.0 kg/m$^2$. High waist-to-hip ratio: waist-to-hip ratio ≥ 0.90 in men and ≥ 0.85 in women. Abdominal obesity: waist circumference > 102 cm in men and > 88 cm in women. *** p < 0.0001; ** p < 0.01; *p < 0.05; ns: not significant p-value. Pool of the following Italian regions: Piedmont, Lombardy, Liguria, Emilia Romagna, Tuscany, Lazio, Abruzzo, Basilicata, Calabria, and Sicily.

**Table S13. Body mass index and measured height, weight, and waist and hip circumferences mean levels by educational level and period (age-adjusted using the European standard population). Italian resident men aged 35–74 years, the CUORE Project Surveys 1998–2002, 2008–2012, and 2018–2019.**

**MEN**

**Body mass index (kg/m²)**

| Educational level | 1998-2002 | | | | ANOVA within period sign | 2008-2012 | | | | ANOVA within period sign | 2018-2019 | | | | ANOVA within period sign | 2018-2019 vs 1998-2002 | | 2018-2019 vs 2008-2012 | |
|---|---|---|---|---|---|---|---|---|---|---|---|---|---|---|---|---|---|---|---|
| | mean | SD | *95% CI* | | *** | mean | SD | *95% CI* | | *** | mean | SD | *95% CI* | | *** | Diff | t-test sign | Diff | t-test sign |
| Higher education | **26,4** | 3,7 | *26,2* | *26,6* | | **27,3** | 4,2 | *27,1* | *27,5* | | **26,7** | 4,0 | *26,4* | *27,0* | | **0,3** | ns | **-0,6** | ** |
| Lower education | **27,0** | 3,8 | *26,8* | *27,2* | | **28,0** | 4,3 | *27,7* | *28,3* | | **27,7** | 4,5 | *27,2* | *28,2* | | **0,7** | * | **-0,3** | ns |

**Height (cm)**

| Educational level | 1998-2002 | | | | ANOVA within period sign | 2008-2012 | | | | ANOVA within period sign | 2018-2019 | | | | ANOVA within period sign | 2018-2019 vs 1998-2002 | | 2018-2019 vs 2008-2012 | |
|---|---|---|---|---|---|---|---|---|---|---|---|---|---|---|---|---|---|---|---|
| | mean | SD | *95% CI* | | *** | mean | SD | *95% CI* | | *** | mean | SD | *95% CI* | | *** | Diff | t-test sign | Diff | t-test sign |
| Higher education | **172,4** | 7,1 | *172,0* | *172,8* | | **173,1** | 6,7 | *172,7* | *173,5* | | **173,8** | 6,5 | *173,4* | *174,3* | | **1,4** | *** | **0,7** | * |
| Lower education | **169,9** | 6,9 | *169,6* | *170,3* | | **170,1** | 6,5 | *169,7* | *170,5* | | **171,2** | 7,0 | *170,5* | *172,0* | | **1,3** | ** | **1,1** | ** |

**Weight (kg)**

| Educational level | 1998-2002 | | | | ANOVA within period sign | 2008-2012 | | | | ANOVA within period sign | 2018-2019 | | | | ANOVA within period sign | 2018-2019 vs 1998-2002 | | 2018-2019 vs 2008-2012 | |
|---|---|---|---|---|---|---|---|---|---|---|---|---|---|---|---|---|---|---|---|
| | mean | SD | *95% CI* | | * | mean | SD | *95% CI* | | ** | mean | SD | *95% CI* | | *ns* | Diff | t-test sign | Diff | t-test sign |
| Higher education | **78,5** | 12,2 | *77,9* | *79,2* | | **81,9** | 14,1 | *81,1* | *82,7* | | **80,8** | 13,1 | *79,8* | *81,7* | | **2,3** | ** | **-1,1** | ns |
| Lower education | **78,0** | 12,3 | *77,4* | *78,6* | | **81,0** | 13,4 | *80,2* | *81,8* | | **81,3** | 14,2 | *79,7* | *82,9* | | **3,3** | ** | **0,3** | ns |

**Waist to Hip circumferences ratio**

| Educational level | 1998-2002 | | | | ANOVA within period sign | 2008-2012 | | | | ANOVA within period sign | 2018-2019 | | | | ANOVA within period sign | 2018-2019 vs 1998-2002 | | 2018-2019 vs 2008-2012 | |
|---|---|---|---|---|---|---|---|---|---|---|---|---|---|---|---|---|---|---|---|
| | mean | SD | *95% CI* | | *** | mean | SD | *95% CI* | | *** | mean | SD | *95% CI* | | *** | Diff | t-test sign | Diff | t-test sign |
| Higher education | **0,93** | 0,06 | *0,93* | *0,94* | | **0,95** | 0,08 | *0,95* | *0,96* | | **0,94** | 0,06 | *0,94* | *0,95* | | **0,01** | *** | **-0,01** | ** |
| Lower education | **0,95** | 0,06 | *0,94* | *0,95* | | **0,97** | 0,07 | *0,96* | *0,97* | | **0,97** | 0,07 | *0,96* | *0,97* | | **0,02** | *** | **0,00** | ns |

**Waist circumferences (cm)**

| Educational level | 1998-2002 | | | | ANOVA within period sign | 2008-2012 | | | | ANOVA within period sign | 2018-2019 | | | | ANOVA within period sign | 2018-2019 vs 1998-2002 | | 2018-2019 vs 2008-2012 | |
|---|---|---|---|---|---|---|---|---|---|---|---|---|---|---|---|---|---|---|---|
| | mean | SD | *95% CI* | | *** | mean | SD | *95% CI* | | *** | mean | SD | *95% CI* | | *** | Diff | t-test sign | Diff | t-test sign |
| Higher education | **94,4** | 10,4 | *93,8* | *94,9* | | **96,3** | 11,7 | *95,6* | *97,0* | | **95,2** | 10,5 | *94,5* | *96,0* | | **0,8** | ns | **-1,1** | * |
| Lower education | **95,4** | 10,7 | *94,9* | *95,9* | | **97,6** | 11,6 | *96,9* | *98,3* | | **97,6** | 11,8 | *96,3* | *98,9* | | **2,2** | ** | **0,0** | ns |

**Hip circumferences (cm)**

| Educational level | 1998-2002 | | | | ANOVA within period sign | 2008-2012 | | | | ANOVA within period sign | 2018-2019 | | | | ANOVA within period sign | 2018-2019 vs 1998-2002 | | 2018-2019 vs 2008-2012 | |
|---|---|---|---|---|---|---|---|---|---|---|---|---|---|---|---|---|---|---|---|
| | mean | SD | *95% CI* | | *ns* | mean | SD | *95% CI* | | *ns* | mean | SD | *95% CI* | | *ns* | Diff | t-test sign | Diff | t-test sign |
| Higher education | **101,2** | 8,5 | *100,7* | *101,7* | | **100,9** | 9,1 | *100,4* | *101,5* | | **100,7** | 7,5 | *100,2* | *101,3* | | **-0,5** | ns | **-0,2** | ns |
| Lower education | **100,9** | 8,7 | *100,5* | *101,3* | | **100,7** | 8,3 | *100,2* | *101,2* | | **100,9** | 8,8 | *99,9* | *101,9* | | **0,0** | ns | **0,2** | ns |

Higher education—high school or university; lower education—primary or middle school. SD: standard deviation; CI: confidence interval; BMI: body mass index. Means and standard deviations were age-standardized using the European Standard Population 2013. ANOVA: Analysis of Variance was used to compare variables between educational levels within periods. t-test was used to compare variables between periods. *** p < 0.0001; ** p < 0.01; *p < 0.05; ns: not significant p-value. 1998–2002 number of men in the higher and lower class of education: 1249 and 1727. 2008–2012 number of men in the higher and lower class of education: 1186 and 1020. 2018–2019 number of men in the higher and lower class of education: 728 and 304. Pool of the following Italian regions: Piedmont, Lombardy, Liguria, Emilia Romagna, Tuscany, Lazio, Abruzzo, Basilicata, Calabria, and Sicily.

**Table S14. Body mass index and measured height, weight, and waist and hip circumferences mean levels by educational level and period (age-adjusted using the European standard population). Italian resident women aged 35–74 years, the CUORE Project Surveys 1998–2002, 2008–2012, and 2018–2019**.

## WOMEN

### Body mass index (kg/m$^2$)

| Educational level | 1998-2002 | | | | ANOVA within period sign | 2008-2012 | | | | ANOVA within period sign | 2018-2019 | | | | ANOVA within period sign | 2018-2019 vs 1998-2002 | | 2018-2019 vs 2008-2012 | |
|---|---|---|---|---|---|---|---|---|---|---|---|---|---|---|---|---|---|---|---|
| | mean | SD | *95% CI* | | *** | mean | SD | *95% CI* | | *** | mean | SD | *95% CI* | | *** | Diff | t-test *sign* | Diff | t-test *sign* |
| Higher education | **25,1** | 4,2 | *24,9* | *25,4* | | **25,6** | 5,0 | *25,4* | *25,9* | | **25,4** | 5,0 | *25,0* | *25,7* | | **0,3** | ns | **-0,2** | ns |
| Lower education | **27,0** | 5,1 | *26,8* | *27,2* | | **27,9** | 5,5 | *27,6* | *28,3* | | **28,3** | 5,6 | *27,7* | *28,9* | | **1,3** | *** | **0,4** | ns |

### Height (cm)

| Educational level | 1998-2002 | | | | ANOVA within period sign | 2008-2012 | | | | ANOVA within period sign | 2018-2019 | | | | ANOVA within period sign | 2018-2019 vs 1998-2002 | | 2018-2019 vs 2008-2012 | |
|---|---|---|---|---|---|---|---|---|---|---|---|---|---|---|---|---|---|---|---|
| | mean | SD | *95% CI* | | *** | mean | SD | *95% CI* | | *** | mean | SD | *95% CI* | | *** | Diff | t-test *sign* | Diff | t-test *sign* |
| Higher education | **159,6** | 6,3 | *159,2* | *159,9* | | **159,2** | 6,0 | *158,9* | *159,6* | | **159,8** | 5,8 | *159,4* | *160,2* | | **0,2** | ns | **0,6** | * |
| Lower education | **157,3** | 6,2 | *157,0* | *157,6* | | **156,7** | 6,4 | *156,3* | *157,1* | | **157,6** | 6,5 | *156,9* | *158,3* | | **0,3** | ns | **0,9** | * |

### Weight (kg)

| Educational level | 1998-2002 | | | | ANOVA within period sign | 2008-2012 | | | | ANOVA within period sign | 2018-2019 | | | | ANOVA within period sign | 2018-2019 vs 1998-2002 | | 2018-2019 vs 2008-2012 | |
|---|---|---|---|---|---|---|---|---|---|---|---|---|---|---|---|---|---|---|---|
| | mean | SD | *95% CI* | | *** | mean | SD | *95% CI* | | *** | mean | SD | *95% CI* | | *** | Diff | t-test *sign* | Diff | t-test *sign* |
| Higher education | **63,8** | 10,8 | *63,1* | *64,4* | | **64,9** | 12,6 | *64,2* | *65,6* | | **64,7** | 12,9 | *63,7* | *65,6* | | **0,9** | ns | **-0,2** | ns |
| Lower education | **66,7** | 12,7 | *66,1* | *67,2* | | **68,4** | 13,2 | *67,6* | *69,2* | | **70,2** | 14,2 | *68,7* | *71,8* | | **3,5** | *** | **1,8** | * |

### Waist to Hip circumferences ratio

| Educational level | 1998-2002 | | | | ANOVA within period sign | 2008-2012 | | | | ANOVA within period sign | 2018-2019 | | | | ANOVA within period sign | 2018-2019 vs 1998-2002 | | 2018-2019 vs 2008-2012 | |
|---|---|---|---|---|---|---|---|---|---|---|---|---|---|---|---|---|---|---|---|
| | mean | SD | *95% CI* | | *** | mean | SD | *95% CI* | | *** | mean | SD | *95% CI* | | *** | Diff | t-test *sign* | Diff | t-test *sign* |
| Higher education | **0,82** | 0,06 | *0,82* | *0,83* | | **0,84** | 0,08 | *0,84* | *0,85* | | **0,83** | 0,07 | *0,82* | *0,83* | | **0,01** | ns | **-0,01** | *** |
| Lower education | **0,84** | 0,06 | *0,84* | *0,85* | | **0,86** | 0,08 | *0,85* | *0,86* | | **0,85** | 0,07 | *0,84* | *0,86* | | **0,01** | ns | **-0,02** | ns |

### Waist circumferences (cm)

| Educational level | 1998-2002 | | | | ANOVA within period sign | 2008-2012 | | | | ANOVA within period sign | 2018-2019 | | | | ANOVA within period sign | 2018-2019 vs 1998-2002 | | 2018-2019 vs 2008-2012 | |
|---|---|---|---|---|---|---|---|---|---|---|---|---|---|---|---|---|---|---|---|
| | mean | SD | *95% CI* | | *** | mean | SD | *95% CI* | | *** | mean | SD | *95% CI* | | *** | Diff | t-test *sign* | Diff | t-test *sign* |
| Higher education | **81,5** | 10,3 | *80,9* | *82,1* | | **84,5** | 12,0 | *83,8* | *85,2* | | **83,6** | 12,1 | *82,7* | *84,5* | | **2,1** | ** | **-0,9** | ns |
| Lower education | **85,9** | 12,0 | *85,4* | *86,5* | | **88,9** | 13,1 | *88,1* | *89,7* | | **90,1** | 13,3 | *88,7* | *91,5* | | **4,2** | *** | **1,2** | ns |

### Hip circumferences (cm)

| Educational level | 1998-2002 | | | | ANOVA within period sign | 2008-2012 | | | | ANOVA within period sign | 2018-2019 | | | | ANOVA within period sign | 2018-2019 vs 1998-2002 | | 2018-2019 vs 2008-2012 | |
|---|---|---|---|---|---|---|---|---|---|---|---|---|---|---|---|---|---|---|---|
| | mean | SD | *95% CI* | | *** | mean | SD | *95% CI* | | *** | mean | SD | *95% CI* | | *** | Diff | t-test *sign* | Diff | t-test *sign* |
| Higher education | **98,8** | 9,8 | *98,2* | *99,3* | | **100,4** | 10,5 | *99,8* | *101,0* | | **100,9** | 10,2 | *100,2* | *101,7* | | **2,1** | *** | **0,5** | ns |
| Lower education | **101,7** | 11,4 | *101,2* | *102,2* | | **103,5** | 11,1 | *102,8* | *104,1* | | **105,6** | 11,3 | *104,4* | *106,8* | | **3,9** | *** | **2,1** | ** |

Higher education—high school or university; lower education—primary or middle school. 1998–2002 number of women in the higher and lower class of education: 1050 and 1878. 2008–2012 number of women in the higher and lower class of education: 1153 and 1014. 2018–2019 number of women in the higher and lower class of education: 722 and 341. CI: confidence interval. Means and standard deviations were age-standardized using the European Standard Population 2013. ANOVA: Analysis of Variance was used to compare variables between educational levels within periods. t-test was used to compare variables between periods. *** p < 0.0001; ** p < 0.01; *p < 0.05; ns not significant p-value. Pool of the following Italian regions: Piedmont, Lombardy, Liguria, Emilia Romagna, Tuscany, Lazio, Abruzzo, Basilicata, Calabria, and Sicily.

**Table S15. Prevalence of body mass index and waist and hip circumferences classes based on measurements, by educational level and period (age-adjusted using the European standard population). Italian resident men aged 35–74 years, the CUORE Project Surveys 1998–2002, 2008–2012, and 2018–2019.**

## MEN

### Normal weight

| Educational level | 1998-2002 % | 95% CI | | Chi-squared test within period sign | 2008-2012 % | 95% CI | | Chi-squared test within period sign | 2018-2019 % | 95% CI | | Chi-squared test within period sign | 2018-2019 vs 1998-2002 Diff | chi-squared sign | 2018-2019 vs 2008-2012 Diff | chi-squared sign |
|---|---|---|---|---|---|---|---|---|---|---|---|---|---|---|---|---|
| | | | | ** | | | | * | | | | ** | | | | |
| Higher education | 36 | 34 | 39 | | 30 | 27 | 33 | | 37 | 33 | 40 | | 1 | *** | 7 | ** |
| Lower education | 30 | 28 | 32 | | 26 | 23 | 29 | | 27 | 22 | 32 | | -3 | *** | 1 | ns |

### Overweight

| Educational level | 1998-2002 % | 95% CI | | Chi-squared test within period sign | 2008-2012 % | 95% CI | | Chi-squared test within period sign | 2018-2019 % | 95% CI | | Chi-squared test within period sign | 2018-2019 vs 1998-2002 Diff | chi-squared sign | 2018-2019 vs 2008-2012 Diff | chi-squared sign |
|---|---|---|---|---|---|---|---|---|---|---|---|---|---|---|---|---|
| | | | | ns | | | | ns | | | | ns | | | | |
| Higher education | 49 | 46 | 51 | | 48 | 45 | 51 | | 46 | 42 | 49 | | -3 | ns | -2 | ns |
| Lower education | 50 | 47 | 52 | | 46 | 43 | 50 | | 47 | 41 | 52 | | -3 | ns | 1 | ns |

### Obesity

| Educational level | 1998-2002 % | 95% CI | | Chi-squared test within period sign | 2008-2012 % | 95% CI | | Chi-squared test within period sign | 2018-2019 % | 95% CI | | Chi-squared test within period sign | 2018-2019 vs 1998-2002 Diff | chi-squared sign | 2018-2019 vs 2008-2012 Diff | chi-squared sign |
|---|---|---|---|---|---|---|---|---|---|---|---|---|---|---|---|---|
| | | | | ** | | | | ** | | | | ** | | | | |
| Higher education | 14 | 12 | 16 | | 22 | 19 | 24 | | 17 | 15 | 20 | | 3 | ns | -5 | * |
| Lower education | 20 | 18 | 21 | | 27 | 25 | 30 | | 25 | 20 | 30 | | 5 | * | -2 | ns |

### High waist-to-hip ratio

| Educational level | 1998-2002 % | 95% CI | | Chi-squared test within period sign | 2008-2012 % | 95% CI | | Chi-squared test within period sign | 2018-2019 % | 95% CI | | Chi-squared test within period sign | 2018-2019 vs 1998-2002 Diff | chi-squared sign | 2018-2019 vs 2008-2012 Diff | chi-squared sign |
|---|---|---|---|---|---|---|---|---|---|---|---|---|---|---|---|---|
| | | | | ** | | | | ** | | | | ** | | | | |
| Higher education | 78 | 75 | 80 | | 80 | 78 | 83 | | 76 | 73 | 79 | | -2 | ns | -4 | * |
| Lower education | 83 | 81 | 84 | | 86 | 84 | 88 | | 84 | 80 | 88 | | 1 | ns | -2 | ns |

### Abdominal obesity

| Educational level | 1998-2002 % | 95% CI | | Chi-squared test within period sign | 2008-2012 % | 95% CI | | Chi-squared test within period sign | 2018-2019 % | 95% CI | | Chi-squared test within period sign | 2018-2019 vs 1998-2002 Diff | chi-squared sign | 2018-2019 vs 2008-2012 Diff | chi-squared sign |
|---|---|---|---|---|---|---|---|---|---|---|---|---|---|---|---|---|
| | | | | ** | | | | ** | | | | ** | | | | |
| Higher education | 22 | 20 | 24 | | 30 | 27 | 32 | | 23 | 20 | 26 | | 1 | ns | -7 | ** |
| Lower education | 27 | 25 | 29 | | 35 | 32 | 38 | | 32 | 27 | 37 | | 5 | ns | -3 | ns |

Higher education—high school or university; lower education—primary or middle school. Prevalence were age-standardized using the European Standard Population 2013. Chi-squared test was used to compare prevalence among periods and among educational levels within the period. Normal weight: 18.5 ≤ body mass index < 25.0 kg/$m^2$. Overweight: 25.0 ≤ body mass index < 30.0 kg/$m^2$. Obesity: body mass index ≥ 30.0 kg/$m^2$. High waist-to-hip ratio: waist-to-hip ratio ≥ 0.90 in men and ≥ 0.85 in women. Abdominal obesity: waist circumference > 102 cm in men and > 88 cm in women. *** p < 0.0001; ** p < 0.01; *p < 0.05; ns: not significant p-value. 1998–2002 number of men in the higher and lower class of education: 1249 and 1727. 2008–2012 number of men in the higher and lower class of education: 1186 and 1020. 2018–2019 number of men in the higher and lower class of education: 728 and 304. Pool of the following Italian regions: Piedmont, Lombardy, Liguria, Emilia Romagna, Tuscany, Lazio, Abruzzo, Basilicata, Calabria, and Sicily.

**Table S16. Prevalence of body mass index and waist and hip circumferences classes based on measurements, by educational level and period (age-adjusted using the European standard population). Italian resident women aged 35–74 years, the CUORE Project Surveys 1998–2002, 2008–2012, and 2018–2019.**

## WOMEN

### Normal weight

| Educational level | 1998-2002 % | 95% CI | | Chi-squared test within period sign | 2008-2012 % | 95% CI | | Chi-squared test within period sign | 2018-2019 % | 95% CI | | Chi-squared test within period sign | 2018-2019 vs 1998-2002 Diff | chi-squared sign | 2018-2019 vs 2008-2012 Diff | chi-squared sign |
|---|---|---|---|---|---|---|---|---|---|---|---|---|---|---|---|---|
| | | | | *** | | | | *** | | | | *** | | | | |
| Higher education | **54** | *51* | *58* | | **51** | *48* | *54* | | **54** | *51* | *58* | | 0 | ns | 3 | ns |
| Lower education | **38** | *35* | *40* | | **34** | *31* | *37* | | **27** | *22* | *32* | | -11 | ** | -7 | ns |

### Overweight

| Educational level | 1998-2002 % | 95% CI | | Chi-squared test within period sign | 2008-2012 % | 95% CI | | Chi-squared test within period sign | 2018-2019 % | 95% CI | | Chi-squared test within period sign | 2018-2019 vs 1998-2002 Diff | chi-squared sign | 2018-2019 vs 2008-2012 Diff | chi-squared sign |
|---|---|---|---|---|---|---|---|---|---|---|---|---|---|---|---|---|
| | | | | ** | | | | ns | | | | *** | | | | |
| Higher education | **29** | *27* | *32* | | **32** | *29* | *34* | | **25** | *21* | *28* | | -4 | * | -7 | *** |
| Lower education | **36** | *34* | *38* | | **34** | *31* | *37* | | **38** | *33* | *43* | | 2 | ns | 4 | *** |

### Obesity

| Educational level | 1998-2002 % | 95% CI | | Chi-squared test within period sign | 2008-2012 % | 95% CI | | Chi-squared test within period sign | 2018-2019 % | 95% CI | | Chi-squared test within period sign | 2018-2019 vs 1998-2002 Diff | chi-squared sign | 2018-2019 vs 2008-2012 Diff | chi-squared sign |
|---|---|---|---|---|---|---|---|---|---|---|---|---|---|---|---|---|
| | | | | *** | | | | *** | | | | *** | | | | |
| Higher education | **14** | *12* | *16* | | **16** | *14* | *18* | | **19** | *16* | *21* | | 5 | ** | 3 | ns |
| Lower education | **25** | *23* | *27* | | **32** | *29* | *34* | | **33** | *28* | *38* | | 8 | ** | 1 | ns |

### High waist-to-hip ratio

| Educational level | 1998-2002 % | 95% CI | | Chi-squared test within period sign | 2008-2012 % | 95% CI | | Chi-squared test within period sign | 2018-2019 % | 95% CI | | Chi-squared test within period sign | 2018-2019 vs 1998-2002 Diff | chi-squared sign | 2018-2019 vs 2008-2012 Diff | chi-squared sign |
|---|---|---|---|---|---|---|---|---|---|---|---|---|---|---|---|---|
| | | | | *** | | | | *** | | | | *** | | | | |
| Higher education | **35** | *32* | *38* | | **44** | *41* | *47* | | **36** | *32* | *39* | | 1 | ns | -8 | * |
| Lower education | **49** | *46* | *51* | | **56** | *53* | *59* | | **51** | *46* | *57* | | 2 | ns | -5 | ns |

### Abdominal obesity

| Educational level | 1998-2002 % | 95% CI | | Chi-squared test within period sign | 2008-2012 % | 95% CI | | Chi-squared test within period sign | 2018-2019 % | 95% CI | | Chi-squared test within period sign | 2018-2019 vs 1998-2002 Diff | chi-squared sign | 2018-2019 vs 2008-2012 Diff | chi-squared sign |
|---|---|---|---|---|---|---|---|---|---|---|---|---|---|---|---|---|
| | | | | *** | | | | *** | | | | *** | | | | |
| Higher education | **26** | *23* | *29* | | **36** | *33* | *39* | | **33** | *30* | *37* | | 7 | ** | -3 | ns |
| Lower education | **41** | *39* | *43* | | **53** | *50* | *56* | | **53** | *48* | *59* | | 12 | *** | 0 | ns |

Higher education—high school or university; lower education—primary or middle school. 1 Prevalence were age-standardized using the European Standard Population 2013. Chi-squared test was used to compare prevalence among periods and among educational levels within the period. Normal weight: 18.5 ≤ body mass index < 25.0 kg/m$^2$. Overweight: 25.0 ≤ body mass index < 30.0 kg/m$^2$. Obesity: body mass index ≥ 30.0 kg/m$^2$. High waist-to-hip ratio: waist-to-hip ratio ≥ 0.90 in men and ≥ 0.85 in women. Abdominal obesity: waist circumference > 102 cm in men and > 88 cm in women. *** $p < 0.0001$; ** $p < 0.01$; *$p < 0.05$; ns not significant p-value. 998–2002 number of women in the higher and lower class of education: 1050 and 1878. 2008–2012 number of women in the higher and lower class of education: 1153 and 1014. 2018–2019 number of women in the higher and lower class of education: 722 and 341.Pool of the following Italian regions: Piedmont, Lombardy, Liguria, Emilia Romagna, Tuscany, Lazio, Abruzzo, Basilicata, Calabria, and Sicily.
